# Strategic decision making in live streaming e-commerce through tripartite evolutionary game analysis

**DOI:** 10.1371/journal.pone.0305427

**Published:** 2024-07-10

**Authors:** Yifan Xu, Jingyu Qi, Jiahao Kong, Weisi Zhang

**Affiliations:** 1 School of Management, Fudan University, Shanghai, China; 2 Logistics Research Center, Shanghai Maritime University, Shanghai, China; Harbin Institute of Technology, CHINA

## Abstract

This article delves into the current popular phenomenon of live streaming e-commerce, with a specific focus on issues related to product quality and after-sales service. It constructs an evolutionary game model that encompasses three key stakeholders: e-commerce platforms, consumers, and streamers. The study conducts a thorough analysis of the interactions and strategic choices among these entities, investigating the stability of equilibrium strategy combinations within the game system and the influence of various factors on decision-making behaviors. Furthermore, the validity of the analytical conclusion is corroborated through the application of simulation analysis methods. The study finds that for the consumer, strategies such as reducing losses encountered due to quality issues under strict demands, enhancing compensation in these scenarios, and increasing benefits for maintaining stringent requirements during live streaming sessions can motivate them to adopt more stringent strategies. For the streamer, essential factors in promoting the selection of high-quality products include increasing the benefits associated with such choices and reducing the probability of quality issues, or alternatively, decreasing the gains from lower-quality selections and increasing the likelihood of encountering quality problems with these products. For the e-commerce platform, strategically adjusting the profit-sharing ratio to maintain collaborative momentum and influence the enthusiasm of both consumers and streamers is a critical strategy to avert market scenarios akin to prisoner’s dilemmas and tragic outcomes. Overall, this research offers profound insights into the complex strategic evolution within the live commerce market, providing valuable guidance for interaction strategies among e-commerce platforms, consumers, and streamers. Its implications for practical decision-making optimization and strategic formulation are of significant importance.

## 1. Introduction

With the widespread application of the internet and mobile devices, live streaming on e-commerce platforms is gaining increasing popularity. In this setup, streamers sell products through real-time interactions while consumers make purchases during the live streaming. This model has gained favor among numerous businesses due to its strong interactivity and notable effectiveness in driving sales. Consumers are also showing increasing recognition and acceptance of this new marketing approach [1,2]. The outbreak of the COVID-19 pandemic further propelled the development of the online live streaming e-commerce marketing industry. As of June 2022, the number of e-commerce live streaming users in China reached 469 million, an increase of 5.33 million since December 2021, accounting for 44.6% of the total internet users. Corresponding to this large user base is the rise of numerous live streaming e-commerce platforms. Among these, Taobao Live, as one of the early entrants in this field, has firmly secured its leading position due to its complete live streaming e-commerce industrial chain. The surge in short-video platforms has also seen the rapid rise of Douyin and Kuaishou, carving out significant market shares alongside Taobao Live. In 2021, Taobao Live reported over 464 million users, a year-over-year increase of 19.5%. Of these, 66.2% viewed and placed orders, with the number of products listed on the platform growing by 53.0%, and the number of transactions increasing by 16.6%. Douyin and Kuaishou exceeded 75 million live streaming e-commerce sessions, a 100% increase compared to the previous year, with the number of live streaming e-commerce product links surpassing 390 million, a 308% increase. Concurrently, as the live streaming e-commerce model rapidly developed, the role of streamers gradually emerged as a new profession, with several "internet celebrity streamers" gaining fame in the process. Taobao’s data shows that on October 20, 2021, the first day of the pre-sale for Double Eleven, Li Jiaqi live streaming for 12 hours and 26 minutes, achieving sales of 11.54 billion yuan, a 198% increase compared to the 3.87 billion yuan on the first day of last year’s pre-sale, nearly tripling in value. Weiya live streaming for 14 hours and 28 minutes, with first-day sales reaching 8.53 billion yuan, a 63% increase from the previous year. Together, these two streamers generated over 20 billion yuan in sales in a single day. In stark contrast, during the 618-shopping festival in 2022, the absence of top streamers like Xueli, Weiya, and Li Jiaqi led to an overall decline in Taobao Live’s transaction volumes, with even two core categories experiencing negative growth, highlighting the significant impact these leading streamers have on sales volumes on live streaming e-commerce platforms.

However, amidst its rapid development, the live streaming e-commerce industry is also fraught with chaos. Issues such as the sale of substandard products, slow and difficult after-sales service have increasingly come under scrutiny [3,4]. In the realm of livestreaming commerce, a spectrum of quality issues has emerged, ranging from Luo Yonghao’s sale of counterfeit wool sweaters to the distribution of fake "bird’s nests" by Kuaishou’s prominent streamer Xinba, which were ultimately revealed to be merely "sugar water." Additionally, there have been numerous consumer grievances concerning malfunctioning air fryers sold by Li Jiaqi, which ceased to function after minimal use. Moreover, the livestreaming sales industry has faced significant after-sales service challenges. A case in point occurred in October 2022, when livestream host Viya promoted a women’s fashion line, leading to consumer complaints regarding the inferior quality and inaccurate sizing of products, coupled with tardy responses to requests for returns or exchanges. Similarly, in May 2023, beauty influencer Li Jiaqi endorsed a facial cream that induced severe allergic reactions in some consumers. Efforts to secure refunds or compensation were met with a lack of empathy from customer service, thereby intensifying consumer discontent and frustration. On November 20, 2021, the China Consumers Association released an analysis report on consumer rights protection public opinion during "Double 11". During the 24-day monitoring period from October 20 to November 12, a total of 21,353,081 consumer rights protection-related messages were collected, averaging about 890,000 messages per day. In the report, star streamers like Li Jiaqi were specifically mentioned for selling products of poor quality and offering inadequate after-sales service during the "Double 11" live streaming. Additionally, a joint report on consumer rights protection public opinion in live streaming e-commerce (2021), indicated that 32.35% of the live streaming e-commerce consumer rights protection public opinion messages involved product quality issues, ranking first.

The occurrence of these incidents can be attributed to multiple factors, such as inadequate clarity in relevant laws and regulations, cautious and tolerant regulation of new business models by regulatory authorities, lack of responsibility on some platforms, and lack of integrity and self-discipline among some streamers. Opportunistic businesses and illegal elements often take chances, seeking economic benefits through borderline illegal activities or at the expense of consumer rights [5,6]. Regarding the quality scandals in celebrity live streaming, e-commerce platforms providing the service should bear the responsibility and obligation of oversight. If consumers cannot seek redress from the merchants for goods purchased on the platform, the platform should also bear joint responsibility [7–9]. However, in such incidents, platforms often manage to perfectly evade visibility, failing to shoulder their due responsibilities, thereby creating further inconvenience for consumer rights protection. Moreover, the nature of live streaming e-commerce itself carries a certain level of concealment, making it difficult to gather evidence.

Consumers often find it challenging to demand after-sales solutions from the streamers or merchants, resulting in a low probability of successful self-initiated rights protection, issues in live streaming e-commerce such as lagging legal systems, distorted regulation, and obstacles in dispute resolution mechanisms, along with consumers’ limited understanding, significantly impact their rights [10–12]. Thus, spreading awareness about consumer rights and providing effective communication tools are vital. Consumer organizations should promote public interest litigation for consumer civil rights, thereby enhancing consumers’ self-help capabilities [13]. In April 2021, Chinese authorities issued the "Management Measures for Online Live Marketing (Trial)", prohibiting fake traffic and counterfeit sales, and advocating for standardized operations in live streaming rooms. With the increase of product categories, government regulation alone is not enough. Some scholars advocate the introduction of third-party management mechanisms and information systems, and the government ensures that these parties fulfill their responsibilities[14,15].

Against the backdrop of the growing trend in live streaming e-commerce, product quality and after-sales service issues have surged as critical concerns within the industry. This paper constructs an evolutionary game model that includes e-commerce platforms, streamers, and consumers to investigate the factors influencing streamers’ product selection. The study examines the income of streamers under various product selection strategies, the probability of encountering quality issues, and the impact of e-commerce platforms’ strategic decisions on their profit coefficients and on the strategic decision-making of all parties involved.

The research yields several important conclusions: Firstly, for consumers, implementing measures to lower the costs associated with enforcing strict quality standards, or to increase the consequences of accepting lower standards that lead to quality issues, as well as enhancing the compensations and benefits for both strict and lenient requirements, prove effective in motivating consumers to uphold stricter standards. Secondly, for streamers, the study finds that increasing the incentives for selecting high-quality products and reducing the likelihood of facing quality issues with these choices, or conversely, reducing the benefits and heightening the risks of opting for low-quality products, can effectively guide and encourage streamers towards offering higher quality products. Lastly, for e-commerce platforms, the analysis shows that significantly reducing the profit share in active collaborations can considerably weaken their motivation for such partnerships, adversely affecting both consumers and streamers. This reduction in motivation could potentially lead the live streaming sales market into a situation similar to the prisoner’s dilemma, resulting in negative outcomes for the market.

The structure of our study is as follows. Section 2 reviews the theory and literature. Section 3 introduces the model. Section 4 derives its equilibria. Section 5 performs a numerical simulation and discusses the effects of variables. Section 6 provides the conclusions, implications, and future research.

## 2. Literature review

Our work is closely related to research in live streaming selling, product quality and after-sales service of live streaming e-commerce and evolutionary game in live streaming e-commerce.

### 2.1. Live streaming e-commerce sales

Live streaming e-commerce sales are a new business model. Brands or streamers sell their products through online video streaming. Compared with traditional e-commerce websites, live broadcasting involves an interactive, entertainment and synchronous environment, enabling real-time interaction between streamers and viewers as well as between viewers [16]. The live broadcast delivery mode of platforms such as Taobao in China is in full swing. Some international e-commerce platforms, such as Amazon and Walmart, are also aware of the advantages of live broadcast and have launched their own live commercial activities and platforms. The rapid development of live broadcasting business has brought new opportunities and challenges to academics, businesses and consumers [17].

With the rapid development of the live streaming e-commerce model [18], streamers have gradually become a new emerging profession, and a group of "Influential streamers" have gradually gained fame in this process. Different from traditional celebrity endorsements, the interaction between streamers and consumers in live broadcasts is more "down-to-earth". Therefore, consumers are more willing to make actual purchases due to the "virtual intimate relationship" established with the streamer [19,20]. Considering the creativity and interactivity of the live broadcast, the sense of social presence that consumers get during watching the live broadcast is conceptualized as a personal experience generated by watching the live broadcast [21]. Quasi-social relationships play a crucial role in increasing purchase intentions, and consumers’ perceived utilitarian value and perceived entertainment value indirectly influence their consumption intentions through their trust in the products and sellers [22]. The most prominent advantage of online live marketing lies in the seamless integration of the internet and the personal charm of streamers, with streamers and targeted audiences selecting each other, and consumers’ expectations for the level of live streaming services will further drive the development of streamers [23]. As the relationship between consumers and streamers progresses to different stages, consumers’ psychological contracts will gradually strengthen, which will also encourage them to maintain consumption stickiness [24,25]. From this, we can see that if consumers engage in consumption behaviors with a specific streamer, they may gain emotional benefits, and streamers can also expand their influence and increase their income as a result. Therefore, this study is based on the influence of consumers’ emotional factors on the design of consumer behavior patterns.

### 2.2. Product quality and after-sales service of live streaming e-commerce

In the booming market, high demand has promoted the continuous upgrading of product quality and after-sales service. When streamers have formed a competitive advantage in product quality and after-sales service, it can often attract more consumers’ attention and choice [26]. Similarly, high-quality after-sales service can also reverse the dynamic changes of market demand [27].

However, due to the rapid rise of live broadcast e-commerce, there are several problems to be solved. On the one hand, the phenomenon of false publicity quality occurs frequently. Some of the broadcasters abused their information superiority, perfunctorily selected products and exaggerated the efficacy of the products when selling them to induce consumers to buy [28]. On the other hand, the phenomenon of after-sales shirking responsibility should not be ignored. In the platform e-commerce live broadcast mode, the division of responsibilities is not clear, so streamers and the platform often shirk the publicity and legal responsibilities of selling products to avoid after-sales responsibilities, which seriously undermines the fair competition order of the market [29].

For the internal quality supervision of streamers on e-commerce platforms, researchers like [30] suggest that platforms can control opportunistic behaviors of sellers by establishing a seller reputation mechanism, thereby improving product quality [31–33]. In addition, some platforms ensure the quality and after-sales service of star-brand products by increasing the proportion of self-streaming by platform-brand merchants. A significant development in this regard is celebrity streamer Li Jiaqi’s transparency in product selection, demonstrated by his popular show "Every Girl’s Offer" on Bilibili, which has garnered over ten million views, facilitating direct consumer interaction with merchants and products. However, the self-interest nature of online shopping platforms means they can’t completely eliminate quality risks for consumers [34,35]. Addressing counterfeit sales in e-commerce, high-quality online word-of-mouth is identified as a key solution [36,37]. Government regulatory bodies are advised to innovate in management and guide the establishment of self-regulatory mechanisms for online reputation platforms [38,39], while e-commerce and social media platforms should establish reasonable regulatory accountability mechanisms [40,41]. In view of these problems, this paper discusses the influencing factors of streamers’ selection quality in the live broadcast delivery and how to improve the selection quality.

### 2.3. Evolutionary game in live streaming e-commerce

However, despite the disclosure of numerous internal operations and the advancement of external regulation, the definition of responsibilities in the live streaming e-commerce process often remains unclear. Streamers may simultaneously assume multiple roles such as merchant and advertising spokesperson, and platforms can act both as operators and suppliers. This ambiguity often leads to a situation where streamers and platforms pass responsibility back and forth, making it challenging for consumers and official regulatory bodies to pinpoint specific responsible parties. This not only often puts platforms in a state of regulatory absence but also increases the difficulty for consumers to assert their rights. Wu et al. [42] and Li et al. [43] conducted research on the current practices of "acquaintance deception pricing" and algorithmic price discrimination by e-commerce platforms, respectively. They found that consumer oversight can effectively curb e-commerce platforms from choosing to engage in regulatory violations. Moreover, adopting reasonable incentive measures can reduce the cost of consumer supervision and promote the sustainable development of e-commerce platforms. Li et al. [44] expanded consumer regulatory channels within the regulatory mechanisms of e-commerce platforms and constructed a tripartite evolutionary game model involving e-commerce platforms, the government, and consumers under information asymmetry. This model analyzes the influencing factors and conditions for stable states in the strategic choices and behavior evolution of all parties. Fan et al. [45] highlighted issues such as counterfeit advertising and fraudulent activities arising from insufficient administrative supervision. They proposed a behavioral evolutionary game process involving platforms, consumers, and streamers. Through customer complaint handling systems and emotional monitoring systems, they aim to swiftly resolve customer complaints and predict consumer actions, thereby enhancing consumer regulation and preventing conflicts.

Through literature review, it can be seen that the success of live broadcast, as an innovative business model, largely benefits from the ingenious use and guidance of the consumer psychology of "fan economy". However, with the rapid development of live broadcast business, the quality and after-sales problems of live broadcast delivery still need to be solved. Reviewing the research of many scholars, it is found that to achieve the standardized development of live broadcast e-commerce market, all parties need to cooperate and supervise. Based on the research of these scholars, this paper extends the study to explore how changes in consumer rights protection costs influence consumers’ standards for product quality, and how platform regulatory intensity and consumer quality standards affect the seriousness of streamers’ product quality selection. To investigate the issues mentioned earlier, this paper employs a tripartite evolutionary game model to study the strategy choices and evolution of e-commerce platforms, consumers, and streamers. The tripartite game research method is widely applied in various fields, coordinated regulation of market-oriented rental housing, and is a common method for studying multi-agent systems [46,47].

Through the review of the literature mentioned above, the innovation of this paper primarily lies in three aspects: Firstly, it employs a tripartite evolutionary game approach to analyze the relationships among multiple agents. Compared to previous research methods, this approach better reflects the mutual constraints and influences among e-commerce platforms, streamers, and consumers. It facilitates the exploration of the evolution of each party’s strategic choices under the influence of multiple factors. Secondly, unlike traditional quality issues, live streaming product quality involves a broader range of subjects. Existing research often treats e-commerce platforms and streamers as a single entity to explore the quality regulation models by the government and third-party regulatory bodies. This paper distinguishes between e-commerce platforms and streamers, studying the factors influencing their profit when making strategic choices, both independently and in relation to consumers. Thirdly, the research question of this paper is based on actual phenomena, taking into account the impact of consumer rights protection costs on consumer quality requirements. It also considers how these requirements, influenced by consumer actions, affect the platforms’ punitive measures and, in turn, the subjective product selection behavior of streamers. This perspective helps fill gaps in research on how to improve the quality of product selection in live streaming e-commerce and offers practical guidance for enhancing the quality of product selection in this domain.

## 3. Model assumptions and construction

**Fig 1:**
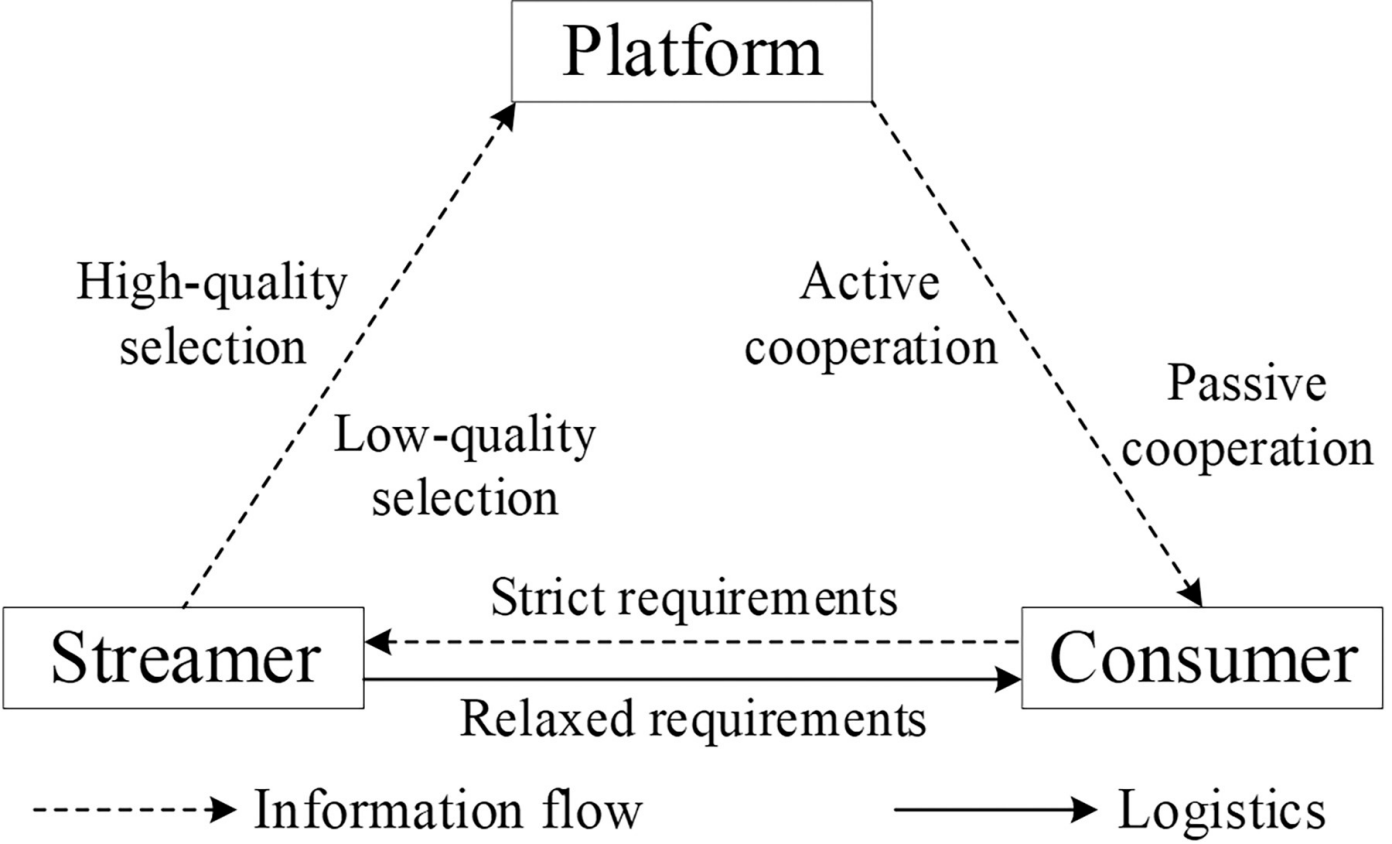
Structure of the tripartite evolutionary strategy model.

Traditional game theory posits that enterprises adhere to specific strategies but fails to examine the long-term evolutionary stability of these strategies under conditions of bounded rationality [48]. Our research delves into the long-term strategic decision-making within the realm of live streaming e-commerce, employing evolutionary game theory to identify evolutionarily stable strategies (ESS) for enterprises over extended periods [49]. Acknowledging the prevalence of bounded rationality in practical scenarios [50], we have developed analytical models grounded in evolutionary game theory. These models take into account the bounded rationality inherent to e-commerce platforms, streamers, and consumers to explore the behavior strategies of enterprises engaged in live streaming e-commerce. In this model, the e-commerce platform profits by collecting commission from the streamer, who gains financially through promoting and selling products. The consumer benefits by purchasing products at relatively advantageous prices. The platform provides a series of services to the streamer, including advertising promotion and technical support, and the brand impact generated by the streamer reciprocates to the platform, boosting sales. The supervisory role of the platform over the streamer is significant, and feedback from the consumer plays a crucial role in the sustainable development of both the streamer and the platform. The strategic evolutionary game relationship among these three entities is depicted in Fig 1.

Based on this model, the analysis explores the evolutionarily stable strategies of various entities in the context of potential quality issues in live streaming e-commerce. It examines the conditions for the existence of stable evolutionary points for all parties involved. Using Matlab 2022a for simulation [51,52], the study analyzes the strategy choices of the three parties in four scenarios. It investigates the loss and compensation received by consumers when quality issues are identified under different requirement conditions, as well as the benefits gained during the live streaming e-commerce viewing process. The research also considers the income of the streamers under different product selection strategies, the probability of quality issues arising, and the impact of different strategic approaches of e-commerce platforms on their profit coefficients on the strategic decision-making of all parties. This analysis provides theoretical support for understanding the systemic evolution of live streaming e-commerce involving three parties. It offers strategic recommendations for the decision-making behaviors of participating entities. Furthermore, the study provides valuable management insights for the future development of the live streaming e-commerce industry and for reducing the impact of quality issues in live streaming e-commerce sold products.

### 3.1. Model assumptions

Assumption 1: In the context of e-commerce live streaming activities, all three participating entities are bounded rationality. The strategies for the e-commerce platform are {Active cooperation, Passive cooperation}, for consumers {Strict requirements, Relaxed requirements}, and for streamers {High-quality selection, Low-quality selection}. In this context, ’High-quality selection’ for live streaming e-commerce streamer involves considering the product’s quality, user experience, and viewer demand for the products showcased in the stream; whereas ’Low-quality selection’ involves restricting their options to simply choosing brands that offer higher commissions.

Assumption 2: Streamers adopting the high-quality product selection strategy earn a profit of *U*_1_, with the probability of encountering quality issues denoted as *w*_1_. Conversely, opting for a low-quality product selection strategy yields a profit of *U*_2_, with the probability of quality issues at *w*_2_. Considering the increased workload associated with a high-quality selection strategy, it is typically assumed that *U*_1_<*U*_2_, as the probability of facing quality problems is lower with high-quality selection, thus *w*_1_<*w*_2_. When quality issues are detected, the loss faced by streamers (comprising compensation and fines levied by the platform) is *F*_*i*_, which is directly proportional to the probability of the product’s quality issues, represented as *F*_*i*_ = *w*_*i*_*f*_*i*_(*i* = 1,2; *j* = 1,2, where *f*_*i*_ corresponds to the amount lost by the e-commerce platform under active and passive cooperation strategies, assuming *f*_1_>*f*_2_.

Assumption 3: The active cooperation of an e-commerce platform involves proactively promoting streamers to the platform’s existing users to expand the streamers’ influence. Passive cooperation, on the other hand, entails only basic promotion, without actively directing traffic to the streamers. The profit coefficient for different cooperation modes is denoted as *r*_*i*_(*i* = 1,2). Considering that active cooperation can increase sales and thus generate more revenue for the platform, it is assumed that *r*_1_>*r*_2_. Furthermore, if quality issues arise with products sold by the streamer team, the platform incurs a loss denoted as *N*_*i*_, and *N*_*j*_ = *w*_*i*_*n*_*j*_(*i* = 1,2; *j* = 1,2). Here, *n*_*j*_ represents the extent of negative impact caused by consumers choosing either strict or relaxed requirements, with the assumption that *n*_1_>*n*_2_. If the platform chooses active cooperation, and quality issues occur in the products sold by the streamer team, the platform will implement certain compensation measures to retain customers who might otherwise leave. The compensations provided to consumers with strict and relaxed requirements are denoted as *E*_1_ and *E*_2_, respectively, with the assumption that *E*_1_>*E*_2_.

Assumption 4: During the live streaming e-commerce viewing process, consumers gain a benefit denoted as *S*_1_ when they have strict requirements for the streamer, and a benefit *S*_2_ under relaxed requirements. Since it is assumed that the streamer has a certain level of popularity, consumers derive emotional benefits when imposing strict requirements, leading to the assumption that *S*_1_>*S*_2_. Additionally, the loss incurred by consumers upon discovering quality issues is *P*_1_ under strict requirements and *P*_2_ under relaxed requirements. Considering that consumers with strict requirements will engage in rights protection activities, thus incurring certain time and economic costs when quality issues arise, it is assumed that *P*_1_>*P*_2_. As the cost incurred by consumers in the accountability process is often not directly proportional to the compensation received, it is assumed that *P*_1_−*P*_2_>*E*_1_−*E*_2_. If consumers have strict requirements for the streamer, and quality issues occur in the products sold, this leads to customer attrition and the associated loss for the streamer is denoted as *A*_*i*_, where *A*_*i*_ = *a*_*i*_*P*_1_(*i* = 1,2). Here, *a*_*i*_ represents the loss for the streamer under the scenarios of high-quality or low-quality selections, with the assumption being *a*_1_<*a*_2_.

The notations used in this model are shown in Table 1.

**Table 1:** Notations for the model.

Notations	Descriptions
*w*_1_	Probability of quality issues with high-quality selection by the streamer
*w*_2_	Probability of quality issues with low-quality selection by the streamer
*U*_1_	Profit for the streamer from high-quality selection strategy
*U*_2_	Profit for the streamer from low-quality selection strategy
*r*_1_	Profit coefficient for the platform under active cooperation
*r*_2_	Profit coefficient for the platform under passive cooperation
*n*_1_	Degree of negative impact under strict consumer requirements
*n*_2_	Degree of negative impact under relaxed consumer requirements
*S*_1_	Revenue for the consumer from strict requirements during live streaming
*S*_2_	Revenue for the consumer from relaxed requirements during live streaming
*P*_1_	Loss to the consumer when quality issues are discovered under strict requirements
*P*_2_	Loss to the consumer when quality issues are discovered under relaxed requirements
*E*_1_	Compensation to the consumer under strict requirements
*E*_2_	Compensation to the consumer under relaxed requirements
*f*_1_	Financial loss to the streamer under active cooperation by the e-commerce platform
*f*_2_	Financial loss to the streamer under passive cooperation by the e-commerce platform
*a*_1_	Loss to the streamer from choosing high-quality selection
*a*_2_	Loss to the streamer from choosing low-quality selection

### 3.2. Model construction

Based on the assumptions and parameters mentioned earlier, the revenue matrix for the e-commerce platform, consumers, and the streamer is as shown in Table 2.

**Table 2:** Strategy combination for e-commerce platform, consumer, and streamer.

Streamer	E-commerce platform			
	Active cooperation (*x*)		Passive cooperation (1−*x*)	
	Consumer			
	Strict requirements (y)	Relaxed requirements (1−*y*)	Strict requirements (*y*)	Relaxed requirements (1−*y*)
High-quality selection (*z*)	(*x*,*y*,*z*)	(*x*,1−*y*,*z*)	(1−*x*,*y*,*z*)	(1−*x*,1−*y*,*z*)
Low-quality selection (1−*z*)	(*x*,*y*,1−*z*)	(*x*,1−*y*,1−*z*)	(1−*x*,*y*,1−*z*)	(1−*x*,1−*y*,1−*z*)

This can be specifically represented in Table 3:

**Table 3:** Revenue matrix for e-commerce platform, consumer, and streamer.

Strategy	E-commerce platform	Consumer	Streamer
(*x*,*y*,*z*)	*r*_1_*U*_1_−*w*_1_*n*_1_−*w*_1_*E*_1_	*S*_1_−*w*_1_*P*_1_+*w*_1_*E*_1_	*U*_1_−*w*_1_*f*_1_−*w*_1_*A*_1_
(*x*,1−*y*,*z*)	*r*_1_*U*_1_−*w*_1_*n*_2_−*w*_1_*E*_2_	*S*_2_−*w*_1_*P*_2_+*w*_1_*E*_2_	*U*_1_−*w*_1_*f*_1_
(1−*x*,*y*,*z*)	*r*_2_*U*_1_−*w*_1_*n*_1_	*S*_1_−*w*_1_*P*_1_	*U*_1_−*w*_2_*f*_2_−*w*_1_*A*_1_
(1−*x*,1−*y*,*z*)	*r*_2_*U*_1_−*w*_1_*n*_2_	*S*_2_−*w*_1_*P*_2_	*U*_1_−*w*_1_*f*_2_
(*x*,*y*,1−*z*)	*r*_1_*U*_2_−*w*_2_*n*_1_−*w*_2_*E*_1_	*S*_1_−*w*_2_*P*_1_+*w*_2_*E*_1_	*U*_2_−*w*_2_*f*_1_−*w*_2_*A*_2_
(*x*,1−*y*,1−*z*)	*r*_1_*U*_2_−*w*_2_*n*_2_−*w*_2_*E*_2_	*S*_2_−*w*_2_*P*_2_+*w*_2_*E*_2_	*U*_2_−*w*_2_*f*_1_
(1−*x*,*y*,1−*z*)	*r*_2_*U*_2_−*w*_2_*n*_1_	*S*_1_−*w*_2_*P*_1_	*U*_2_−*w*_2_*f*_2_−*w*_2_*A*_2_
(1−*x*,1−*y*,1−*z*)	*r*_2_*U*_2_−*w*_2_*n*_2_	*S*_2_−*w*_2_*P*_2_	*U*_2_−*w*_2_*f*_2_

## 4. Analysis of the tripartite evolutionary game model

Building on the preceding assumptions and models, the strategic choices and payoff scenarios of the three parties, namely e-commerce platforms, consumers, and streamers, demonstrate the feasibility of establishing replicator dynamic equations for their strategy selections according to evolutionary game theory. This process results in the formation of a three-dimensional dynamic system. Furthermore, this approach facilitates the analysis of the dynamic changes in the strategy selections of the involved parties, aiming to explore the evolutionary paths and patterns of the market.

### 4.1. Equilibrium points of the tripartite evolutionary game system

Let the expected revenues for the e-commerce platform when choosing active cooperation and passive cooperation be *M*_*x*_ and *M*_1−*x*_ respectively. Denote their average revenue as *M*_*a*_. Then, the following relationship can be established:

$$M_{x}=yz(r_{1}U_{1}-w_{1}n_{1}-w_{1}E_{1})+(1-y)z(r_{1}U_{1}-w_{1}n_{2}-w_{1}E_{2})+y(1-z)(r_{1}U_{2}-w_{2}n_{1}-w_{2}E_{1})+(1-y)(1-z)(r_{1}U_{2}-w_{2}n_{2}-w_{2}E_{2})$$ (1)

$$M_{1-x}=yz(r_{2}U_{1}-w_{1}n_{1})+(1-y)z(r_{2}U_{1}-w_{1}n_{2})+y(1-z)(r_{2}U_{2}-w_{2}n_{1})+(1-y)(1-z)(r_{2}U_{2}-w_{2}n_{2})$$ (2)

$$M_{a}=xM_{x}+(1-x)M_{1-x}$$ (3)

Let the expected revenues for consumers when choosing strict and general requirements be *M*_*y*_ and *M*_1−*y*_ respectively. Denote their average revenue as *M*_*b*_. Then, the following relationship can be established:

$$M_{y}=xz(S_{1}-w_{1}P_{1}+w_{1}E_{1})+(1-x)z(S_{1}-w_{1}P_{1})+x(1-z)(S_{1}-w_{2}P_{1}+w_{2}E_{1})+(1-x)(1-z)(S_{1}-w_{2}P_{1})$$ (4)

$$M_{1-y}=xz(S_{2}-w_{1}P_{2}+w_{1}E_{2})+(1-x)z(S_{2}-w_{1}P_{2})+x(1-z)(S_{2}-w_{2}P_{2}+w_{2}E_{2})+(1-x)(1-z)(S_{2}-w_{2}P_{2})$$ (5)

$$M_{b}=yM_{y}+(1-y)M_{1-y}$$ (6)

Let the expected revenues for the streamer when choosing high-quality and low-quality selections be *M*_*z*_ and *M*_1−*z*_ respectively. Denote their average revenue as *M*_*c*_. Then, the following relationship can be established:

$$M_{z}=xy(U_{1}-w_{1}f_{1}-w_{1}A_{1})+x(1-y)(U_{1}-w_{1}f_{1})+(1-x)y(U_{1}-w_{1}f_{2}-w_{1}A_{1})+(1-x)(1-y)(U_{1}-w_{1}f_{2})$$ (7)

$$M_{1-z}=xy(U_{2}-w_{2}f_{1}-w_{2}A_{2})+x(1-y)(U_{2}-w_{2}f_{1})+(1-x)y(U_{2}-w_{2}f_{2}-w_{2}A_{2})+(1-x)(1-y)(U_{2}-w_{2}f_{2})$$ (8)

$$M_{c}=zM_{z}+(1-z)M_{1-z}$$ (9)

The system, composed of the above dynamic equations, is as follows:

$$\{\begin{array}{l} F(x)=\frac{dx}{dt}=x(M_{x}-M_{a})=x(x-1)(E_{2}w_{2}-U_{2}r_{1}+U_{2}r_{2}+y(E_{1}-E_{2})w_{2}+z(w_{1}-w_{2})E_{2}-z(r_{1}-r_{2})(U_{1}-U_{2})+yz(w_{1}-w_{2})(E_{1}-E_{2}))\\ F(y)=\frac{dy}{dt}=y(M_{y}-M_{b})=-y(y-1)(S_{1}-S_{2}-(zw_{1}+(1-z)w_{2})(P_{1}-P_{2}-E_{1}x+E_{2}x))\\ F(z)=\frac{dz}{dt}=z(M_{z}-M_{c})=-z(z-1)(U_{1}-U_{2}-y(w_{1}A_{1}-w_{2}A_{2})-(w_{1}-w_{2})(xf_{1}+(1-x)f_{2})) \end{array}$$ (10)

### 4.2. Analysis of the evolutionary paths of strategies for each entity

In a differential dynamical system, the initial values *x*_0_,*y*_0_, and *z*_0_ significantly impact the system’s evolutionary outcome. Therefore, by analyzing the three-dimensional dynamical system *M* of the live streaming e-commerce regulatory game market, we can examine the dynamic changes in strategy choices of the three participating entities and explore the market’s evolutionary paths and patterns. Based on the nature of evolutionarily stable strategies and the stability theorem of differential equations, for the differential equation *i*(*t*) = *F*(*i*), where i = {*x*,*y*,*z*}, when the system is in a stable state for a certain strategy, the derivative of the system’s replicator dynamic equation *F*(*i*) must satisfy *F*(*i*) = 0 and *F*′(*i*)<0. For ease of calculation, according to Formula (10), let $y^{*}=\frac{{-zE}_{2}(w_{1}-w_{2})-E_{2}w_{2}+(zU_{1}-zU_{2}+U_{2})(r_{1}-r_{2})}{\left(E_{1}-E_{2})(w_{1}z-w_{2}z+w_{2}\right)},$ $z^{*}=\frac{-w_{2}x(E_{1}-E_{2})+w_{2}(P_{1}-P_{2})-S_{1}+S_{2}}{\left(w_{1}-w_{2})(E_{1}x-E_{2}x-P_{1}+P_{2}\right)},$ $x^{*}=\frac{U_{1}-U_{2}-f_{2}(w_{1}-w_{2})-y(A_{1}w_{1}-A_{2}w_{2})}{\left(f_{1}-f_{2})(w_{1}-w_{2}\right)}$, we can obtain the threshold values y*,z*, and x*, leading to the following proposition.

#### 4.2.1. E-commerce platform

**Proposition 1**: For the e-commerce platform, when *y* = y*, where $y^{*}=\frac{{-zE}_{2}(w_{1}-w_{2})-E_{2}w_{2}+(z(U_{1}-U_{2})+U_{2})(r_{1}-r_{2})}{\left(E_{1}-E_{2})((w_{1}-w_{2})z+w_{2}\right)}$, the platform will not change its initially chosen strategy. When *y*<y*, the platform chooses active collaboration. Conversely, when *y*>y*, the platform switches to passive collaboration.

Proof: Considering that the first derivative of *F*(*x*) with respect to $x,\frac{dF(x)}{dx}=(2x-1)(E_{2}w_{2}-(U_{2}+U_{1}z-U_{2}z)(r_{1}-r_{2})+w_{2}y(E_{1}-E_{2})+E_{2}z(w_{1}-w_{2})+yz(w_{1}-w_{2})(E_{1}-E_{2}))$, let $G(y)=E_{2}w_{2}-(U_{2}+U_{1}z-U_{2}z)(r_{1}-r_{2})+w_{2}y(E_{1}-E_{2})+E_{2}z(w_{1}-w_{2})+yz(w_{1}-w_{2})(E_{1}-E_{2})$. According to the stability theorem of differential equations, for the probability of the e-commerce platform choosing active collaboration to be in a stable state, it must satisfy: *F*(*x*) = 0 and $\frac{dF(x)}{dx}<0$. Given that *E*_1_>*E*_2_,0<*w*_1_<*w*_2_, and *z*∈[0,1], it follows that $\frac{\partial G(y)}{\partial y}=(E_{1}-E_{2})(zw_{1}+(1-z)w_{2})>0$. Thus, *G*(*y*) is an increasing function with respect to *y*. Therefore, when *y* = y*,*F*(*x*) = 0 and $\frac{dF(x)}{dx}=0$, the stable strategy is indeterminate. When *y*<y*,*G*(*y*)<0, satisfying the conditions *F*(*x*) = 0 and $\frac{dF(x)}{dx}<0$, taking *x* = 1 as the Evolutionarily Stable Strategy (ESS) for the e-commerce platform. When *y*>y*,*G*(*y*)>0, the conditions *F*(*x*) = 0 and $\frac{dF(x)}{dx}<0$ are satisfied, taking *x* = 0 as ESS.

**Fig 2:**
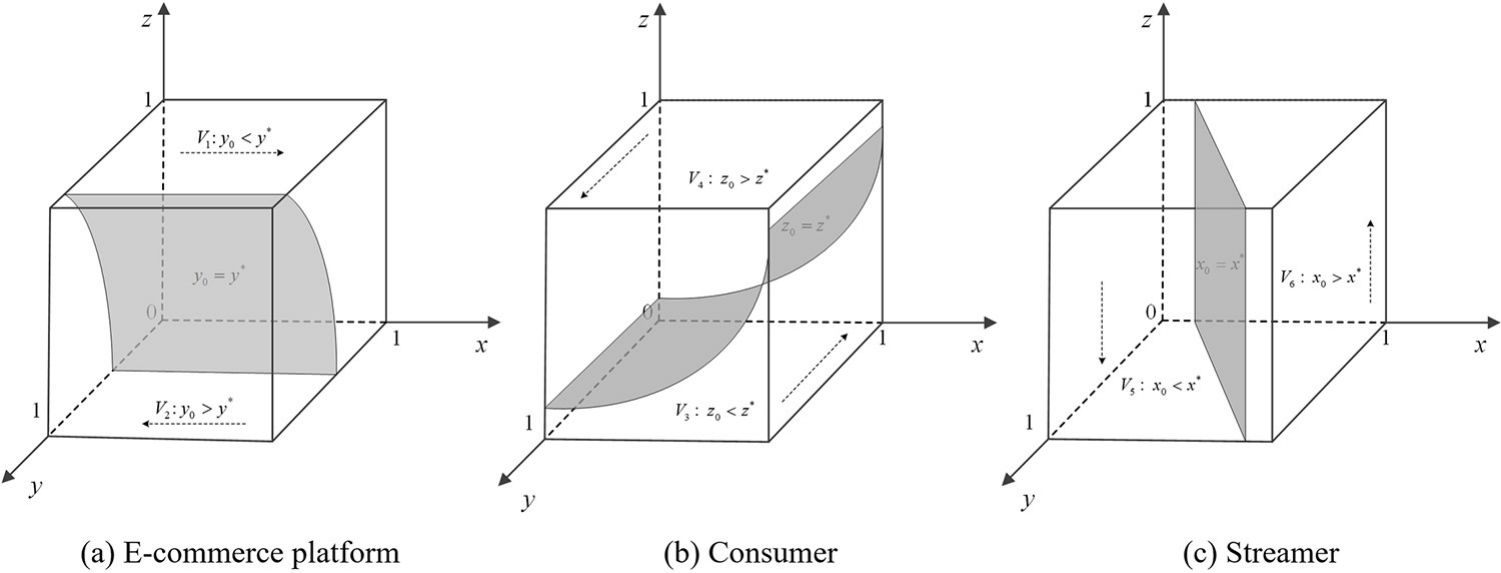
Replicator phase diagram for strategy evolution in live streaming e-commerce.

Based on the above analysis, a replicator phase diagram illustrating the strategy evolution of the e-commerce platform is drawn, as shown in Fig 2(A). From the diagram, it can be seen that when the initial state of the e-commerce platform’s strategy is in space *V*_1_, where 0<*y*<y*,*x* = 1 is the evolutionary strategy equilibrium point. In this state, the e-commerce platform opts for an active collaboration strategy. Conversely, when the initial strategy state of the e-commerce platform is in space *V*_2_, where y*<*y*<1, *x* = 0 is the evolutionary strategy equilibrium point, and the platform selects a passive collaboration strategy.

As indicated in Fig 2(A), when 0<*y*<y*,*x* = 1 is the evolutionary strategy equilibrium point, and the e-commerce platform chooses an active collaboration strategy corresponding to volume *V*_1_. When *y*<y*<1, *x* = 0 is the evolutionary strategy equilibrium point, and the e-commerce platform opts for a passive collaboration strategy corresponding to volume *V*_2_. The volume *V*_1_ is given $V_{1}=\int_{0}^{1}\int_{0}^{1}\frac{{-zE}_{2}(w_{1}-w_{2})-E_{2}w_{2}+(zU_{1}-zU_{2}+U_{2})(r_{1}-r_{2})}{\left(E_{1}-E_{2})(w_{1}z-w_{2}z+w_{2}\right)}dzdx$, which simplifies to $V_{1}=\frac{(w_{1}-w_{2})(\left(r_{1}-r_{2})(U_{1}-U_{2})+E_{2}(-w_{1}+w_{2}\right))+(r_{1}-r_{2})(U_{2}w_{1}-U_{1}w_{2})\log\left(\frac{w_{1}}{w_{2}}\right)}{(E_{1}-E_{2}){\left(w_{1}-w_{2}\right)}^{2}}$, and *V*_2_ is given by *V*_2_ = 1−*V*_1_.

**Corollary 1:** *The probability of an e-commerce platform choosing active collaboration is positively correlated with the revenue from the streamer’s high-quality selection strategy and negatively correlated with the revenue from the streamer’s low-quality selection strategy*. *This correlation also applies to the coefficients of revenue for active and passive collaboration strategies under specific conditions*.

Proof: Based on the expression for the probability of active collaboration *V*_1_ by the e-commerce platform, the first-order partial derivatives of various elements are calculated. For the derivative with respect to $U_{1},\frac{\partial V_{1}}{\partial U_{1}}=\frac{\left(r_{1}-r_{2})(w_{1}-w_{2}-w_{2}\log\left(\frac{w_{1}}{w_{2}}\right)\right)}{(E_{1}-E_{2}){\left(w_{1}-w_{2}\right)}^{2}}$, and for $U_{2},\frac{\partial V_{1}}{\partial U_{2}}=\frac{-(r_{1}-r_{2})(w_{1}-w_{2}-w_{2}\log\left(\frac{w_{1}}{w_{2}}\right))}{(E_{1}-E_{2}){\left(w_{1}-w_{2}\right)}^{2}}$. Similarly, the derivatives with respect to *r*_1_ and *r*_2_ are $\frac{\partial V_{1}}{\partial r_{1}}=\frac{(U_{1}-U_{2})(w_{1}-w_{2})+(U_{2}w_{1}-U_{1}w_{2})\log\left(\frac{w_{1}}{w_{2}}\right)}{(E_{1}-E_{2}){\left(w_{1}-w_{2}\right)}^{2}}$ and $\frac{\partial V_{1}}{\partial r_{2}}=\frac{-(U_{1}-U_{2})(w_{1}-w_{2})-(U_{2}w_{1}-U_{1}w_{2})\log\left(\frac{w_{1}}{w_{2}}\right)}{(E_{1}-E_{2}){\left(w_{1}-w_{2}\right)}^{2}}$, respectively. Defining $\frac{w_{1}}{w_{2}}$ as *x*_*w*_ and considering the function *x*_*w*_−(1+log*x*_*w*_)>0 within the interval *x*_*w*_∈(0,1) implies that this function is positive. Given that *r*_1_>*r*_2_,*E*_1_>*E*_2_,*U*_1_<*U*_2_, and *w*_1_<*w*_2_, it follows that $\frac{\partial V_{1}}{\partial U_{1}}>0$ and $\frac{\partial V_{1}}{\partial U_{2}}<0$. When the conditions $\frac{w_{1}}{w_{2}}<\frac{U_{1}}{U_{2}}$ and *E*_2_(*w*_2_−*w*_1_)<(*r*_1_−*r*_2_)(*U*_2_−*U*_1_) are satisfied, then $\frac{\partial V_{1}}{\partial r_{1}}>0$ and $\frac{\partial V_{1}}{\partial r_{2}}<0$.

Thus, Corollary 1 implies that securing the streamer’s revenue in high-quality product selection can discourage general collaboration by the e-commerce platform. Additionally, when the ratio of revenue from low-quality to high-quality selection by the streamer exceeds the ratio of probabilities of encountering problems $\frac{w_{1}}{w_{2}}<\frac{U_{1}}{U_{2}}$, increasing the coefficient of revenue for active collaboration can encourage the platform to adopt an active collaboration strategy.

#### 4.2.2. Consumers

**Proposition 2:** For consumers, when *z* = z*, where the threshold $z^{*}=\frac{-w_{2}x(E_{1}-E_{2})+w_{2}(P_{1}-P_{2})-S_{1}+S_{2}}{\left(w_{1}-w_{2})(E_{1}x-E_{2}x-P_{1}+P_{2}\right)}$, consumers will not change their initially chosen strategy. When *z*>z*, consumers choose strict requirements; when *z*<z*, they switch their strategy to relaxed requirements.

Proof: Since the first derivative of *F*(*y*) with respect to $y,\frac{dF(y)}{dy}=(1-2y)(S_{1}-S_{2}-(zw_{1}+(1-z)w_{2})(P_{1}-P_{2}-E_{1}x+E_{2}x))$, let $H(z)=S_{1}-S_{2}-(zw_{1}+(1-z)w_{2})(P_{1}-P_{2}-E_{1}x+E_{2}x)$. According to the stability theorem of differential equations, for the probability of consumers choosing strict requirements to be in a stable state, it must satisfy: *F*(*y*) = 0 and $\frac{dF(y)}{dy}<0$. Given that *E*_1_>*E*_2_,0<*w*_1_<*w*_2_,*P*_1_−*P*_2_>*E*_1_−*E*_2_ and *x*∈[0,1], it follows that $\frac{\partial H(z)}{\partial z}=(w_{1}-w_{2})(xE_{1}-xE_{2}+P_{2}-P_{1})>0$, making *H*(*z*) an increasing function with respect to *z*. Therefore, when *z* = z*,*F*(*y*) = 0 and $\frac{dF(y)}{dy}=0$, leaving the stable strategy indeterminate. When *z*<z*,*H*(*z*)<0, satisfying the conditions *F*(*y*) = 0 and $\frac{dF(y)}{dy}<0$, taking *y* = 0 as the Evolutionarily Stable Strategy (ESS) for consumers. When *z*>z*,*H*(*z*)>0, satisfying *F*(*y*) = 0 and $\frac{dF(y)}{dy}<0$, with *y* = 1 as ESS.

Based on the aforementioned analysis, a replicator phase diagram depicting the strategy evolution of consumers is created, as shown in Fig 2(B). The diagram indicates that when the initial state of the consumer strategy is in space *V*_3_, where 0<*z*<z*,*y* = 0 is the evolutionary strategy equilibrium point. In this state, consumers opt for a lenient requirement strategy. Conversely, when the initial state of the consumer strategy is in space *V*_4_, where z*<*z*<1, *y* = 1 is the evolutionary strategy equilibrium point, indicating that consumers choose a strict requirement strategy.

As demonstrated in Fig 2(B), when 0<*z*<z*, with *y* = 0 being the evolutionary strategy equilibrium point, consumers select a lenient requirement strategy, corresponding to volume *V*_3_. When z*<*z*<1, with *y* = 1 as the evolutionary strategy equilibrium point, consumers opt for a strict requirement strategy, corresponding to volume *V*_4_. The volume *V*_3_ is calculated by the integral $V_{3}=\int_{0}^{1}\int_{0}^{1}\frac{-w_{2}x(E_{1}-E_{2})+w_{2}(P_{1}-P_{2})-S_{1}+S_{2}}{\left(w_{1}-w_{2})(E_{1}x-E_{2}x-P_{1}+P_{2}\right)}dxdy$, which simplifies to $V_{3}=(w_{1}-w_{2})(-w_{2}+\frac{(S_{1}-S_{2})\log\left(\frac{P_{2}-P_{1}}{E_{1}-E_{2}-P_{1}+P_{2}}\right)}{\left(E_{1}-E_{2}\right)})$. The volume *V*_4_ is given by *V*_4_ = 1−*V*_3_.

**Corollary 2:** *The probability of consumers choosing relaxed requirements is positively correlated with the loss of strict requirement consumers*, *the gain of relaxed requirement consumers*, *and the compensation received under relaxed requirements*. *It is negatively correlated with the loss of relaxed requirement consumers*, *the gain of strict requirement consumers*, *and the compensation received under strict requirements*.

Proof: Based on the expression for the probability of relaxed requirements *V*_3_, the first-order partial derivatives as: $\frac{\partial V_{3}}{\partial P_{1}}=-\frac{\left(w_{1}-w_{2})(S_{1}-S_{2}\right)}{\left(P_{1}-P_{2})(E_{2}-E_{1}-P_{2}+P_{1}\right)},$ $\frac{\partial V_{3}}{\partial P_{2}}=\frac{\left(w_{1}-w_{2})(S_{1}-S_{2}\right)}{\left(P_{1}-P_{2})(E_{2}-E_{1}-P_{2}+P_{1}\right)},$ $\frac{\partial V_{3}}{\partial E_{1}}=\frac{\left(w_{1}-w_{2})(S_{1}-S_{2})(E_{2}-E_{1}+\log\left(\frac{P_{2}-P_{1}}{E_{1}-E_{2}-P_{1}+P_{2}}\right)(E_{2}-E_{1}-P_{2}+P_{1})\right)}{{\left(E_{1}-E_{2}\right)}^{2}(E_{1}-E_{2}-P_{1}+P_{2})},$ $\frac{\partial V_{3}}{\partial E_{2}}=\frac{\left(w_{1}-w_{2})(S_{1}-S_{2})(\frac{E_{2}-E_{1}}{\left(E_{2}-E_{1}-P_{2}+P_{1}\right)}+log(\frac{P_{2}-P_{1}}{E_{1}-E_{2}-P_{1}+P_{2}})\right)}{{\left(E_{1}-E_{2}\right)}^{2}},$ $\frac{\partial V_{3}}{\partial S_{1}}=\frac{(w_{1}-w_{2})log(\frac{P_{2}-P_{1}}{E_{1}-E_{2}-P_{1}+P_{2}})}{\left(E_{1}-E_{2}\right)},$ $\frac{\partial V_{3}}{\partial S_{2}}=\frac{(w_{2}-w_{1})log(\frac{P_{2}-P_{1}}{E_{1}-E_{2}-P_{1}+P_{2}})}{\left(E_{1}-E_{2}\right)}$, given that *P*_1_>*P*_2_,*S*_1_>*S*_2_,*w*_1_<*w*_2_, and *P*_1_−*P*_2_>*E*_1_−*E*_2_. Therefore, $\frac{\partial V_{3}}{\partial P_{1}}>0,\frac{\partial V_{3}}{\partial E_{2}}>0,\frac{\partial V_{3}}{\partial S_{2}}>0,\frac{\partial V_{1}}{\partial U_{2}}<0,\frac{\partial V_{3}}{\partial E_{1}}<0,\frac{\partial V_{3}}{\partial S_{1}}<0$.

Corollary 2 indicates that e-commerce platforms can encourage consumers to choose strict requirements strategies by reducing consumer rights protection costs or increasing compensation for consumers, subsequently promoting streamers to select high-quality products.

#### 4.2.3. Streamer

**Proposition 3:** For the streamer, when *x* = x*, where the threshold $x^{*}=\frac{U_{1}-U_{2}-f_{2}(w_{1}-w_{2})-y(A_{1}w_{1}-A_{2}w_{2})}{\left(f_{1}-f_{2})(w_{1}-w_{2}\right)}$, the streamer will not change their initially chosen strategy. When *x*<x*, the streamer opts for low-quality selection; when *x*>x*, the streamer changes to high-quality selection.

Proof: Since the first derivative of *F*(*z*) with respect to $z,\frac{dF(z)}{dz}=(1-2z)(U_{1}-U_{2}-y(w_{1}A_{1}-w_{2}A_{2})-(w_{1}-w_{2})(xf_{1}+(1-x)f_{2}))$, let $R(x)=U_{1}-U_{2}-y(w_{1}A_{1}-w_{2}A_{2})-(w_{1}-w_{2})(xf_{1}+(1-x)f_{2})$. According to the stability theorem of differential equations, for the streamer’s strategy to be in a stable state, it must satisfy: *F*(*z*) = 0 and $\frac{dF(z)}{dz}<0$. Given that *E*_1_>*E*_2_,0<*w*_1_<*w*_2_, and *z*∈[0,1], it follows that $\frac{\partial R(x)}{\partial x}=(w_{2}-w_{1})(f_{1}-f_{2})>0$, making *R*(*x*) an increasing function with respect to *x*. Therefore, when *x* = x*,*F*(*x*) = 0 and $\frac{dF(x)}{dx}=0$, leaving the stable strategy indeterminate. When *x*<x*,*R*(*x*)<0, satisfying *F*(*x*) = 0 and $\frac{dF(x)}{dx}<0$, taking *z* = 0 as the Evolutionarily Stable Strategy (ESS) for the streamer. When *x*>x*,*R*(*x*)>0, satisfying *F*(*x*) = 0 and $\frac{dF(x)}{dx}<0$, with *z* = 1 as ESS.

Based on this analysis, a replicator phase diagram for the streamer’s strategy evolution is drawn, as shown in Fig 2(C). The diagram indicates that when the initial state of the streamer’s strategy is in space *V*_5_, where 0<*x*<x*,*z* = 0 is the evolutionary strategy equilibrium point, indicating that the streamer opts for low-quality selection. When the initial state is in space *V*_6_, where *x*>x*, *z* = 1 is the evolutionary strategy equilibrium point, with the streamer choosing high-quality selection. The volume *V*_5_ is calculated as $V_{5}=\int_{0}^{1}\int_{0}^{1}\frac{{-zE}_{2}(w_{1}-w_{2})-E_{2}w_{2}+(zU_{1}-zU_{2}+U_{2})(r_{1}-r_{2})}{\left(E_{1}-E_{2})(w_{1}z-w_{2}z+w_{2}\right)}dydz$, which simplifies to $V_{5}=\frac{\left(w_{1}-w_{2})(2(U_{1}-U_{2})-w_{1}(A_{1}+2f_{2})+w_{2}(A_{2}+2f_{2})\right)}{2(f_{1}-f_{2})}$. The volume *V*_6_ is given by *V*_6_ = 1−*V*_5_.

**Corollary 3:** *The probability of the streamer choosing low-quality selection is positively correlated with the loss incurred from choosing high-quality selection and the revenue from low-quality selection*. *It is negatively correlated with the loss from low-quality selection and the gain of strict requirement consumers*. *Under certain conditions*, *it is positively correlated with the penalty intensity of general collaboration by the e-commerce platform and the probability of quality issues arising in high-quality selection*, *and negatively correlated with the penalty intensity of active collaboration by the platform and the probability of quality issues in low-quality selection*.

Proof: Based on the expression for the probability of low-quality selection *V*_5_, the first-order partial derivatives are calculated as follows: $\frac{\partial V_{5}}{\partial A_{1}}=\frac{w_{1}(w_{2}-w_{1})}{2(f_{1}-f_{2})},\frac{\partial V_{5}}{\partial U_{1}}=\frac{\left(w_{2}-w_{1}\right)}{\left(f_{1}-f_{2}\right)},\frac{\partial V_{5}}{\partial A_{1}}=\frac{w_{2}(w_{1}-w_{2})}{2(f_{1}-f_{2})},\frac{\partial V_{5}}{\partial U_{1}}=\frac{\left(w_{1}-w_{2}\right)}{\left(f_{1}-f_{2}\right)}$. Given that *f*_1_>*f*_2_ and *w*_1_<*w*_2_, it follows that $\frac{\partial V_{5}}{\partial A_{1}}>0,\frac{\partial V_{5}}{\partial U_{2}}>0,\frac{\partial V_{5}}{\partial A_{2}}<0,\frac{\partial V_{5}}{\partial U_{1}}<0$. When the conditions $2(U_{2}-U_{1})+w_{1}(A_{1}+2f_{2})>w_{2}(A_{2}+2f_{2})$ and $2(U_{1}-U_{2}){>2w}_{1}(A_{1}+2f_{2})-w_{2}({A_{1}+A}_{2}+4f_{2})$ are met, $\frac{\partial V_{5}}{\partial f_{2}}>0,\frac{\partial V_{5}}{\partial w_{1}}>0,\frac{\partial V_{5}}{\partial f_{1}}<0,\frac{\partial V_{5}}{\partial w_{2}}<0$.

Corollary 3 indicates that ensuring revenue for the streamer during high-quality selection can prevent the streamer from opting for a low-quality selection strategy. Additionally, the platform can increase penalty intensity to encourage streamers to select high-quality products.

### 4.3. System equilibrium strategies and stability analysis

Setting *F*(*x*) = 0,*F*(*y*) = 0,*F*(*z*) = 0, which signifies no change in the system’s strategy choices, results in 15 equilibrium points. Among these, *M*_1_,*M*_2_,*M*_3_,*M*_4_,*M*_5_,*M*_6_,*M*_7_,*M*_8_ are equilibrium solutions located on the boundary of the tripartite evolutionary game’s equilibrium domain M={(x,y,z)|0≤x≤1,0≤y≤1,0≤z≤1}. Within this domain, there also exist *M*_9_,*M*_10_,*M*_11_,*M*_12_,*M*_13_,*M*_14_,*M*_15_ that satisfy the conditions. Due to the asymmetric game resulting from information asymmetry, the evolutionary stable strategies should be pure strategies. Therefore, only the eight pure strategy local equilibrium points $M_{1}(0,0,0),M_{2}(1,0,0),M_{3}(0,1,0),M_{4}(0,0,1),M_{5}(1,1,0),M_{6}(1,0,1),M_{7}(0,1,1),M_{8}(1,1,1)$ are discussed.

To verify the stability of the equilibrium strategies, the local stability can be determined by solving the system’s Jacobian matrix (*J*) [53,54]. The system’s Jacobian matrix (*J*) can be derived based on Eq (10).


$$J=\left[\begin{array}{ccc} (2x-1)\left(\begin{array}{c} E_{2}w_{2}-U_{2}r_{1}+U_{2}r_{2}+y(E_{1}-E_{2})w_{2}\\ +z(w_{1}-w_{2})E_{2}-z(r_{1}-r_{2})(U_{1}-U_{2})+\\ yz(w_{1}-w_{2})(E_{1}-E_{2}) \end{array}\right) & x(x-1)(E_{1}-E_{2})(zw_{1}+(1-z)w_{2}) & x(x-1)\left(\begin{array}{c} \left(w_{1}-w_{2})(yE_{1}+(1-y)E_{2}\right)\\ -(r_{1}-r_{2})(U_{1}-U_{2}) \end{array}\right)\\ -y(y-1)(zw_{1}+(1-z)w_{2})(E_{1}-E_{2}) & (1-2y)\left(\begin{array}{c} S_{1}-S_{2}-(zw_{1}+(1-z)w_{2})\\ \left(P_{1}-P_{2}-E_{1}x+E_{2}x\right) \end{array}\right) & y(y-1)(w_{1}-w_{2})(P_{1}-P_{2}-E_{1}x+E_{2}x)\\ z(z-1)(w_{1}-w_{2})(f_{1}-f_{2}) & z(z-1)(w_{1}A_{1}-w_{2}A_{2}) & (1-2z)\left(\begin{array}{c} U_{1}-U_{2}-y(w_{1}A_{1}-w_{2}A_{2})\\ -(w_{1}-w_{2})(xf_{1}+(1-x)f_{2}) \end{array}\right) \end{array}\right]$$ (11)

Using the first method of Lyapunov [55,56], the stability of equilibrium points can be determined by analyzing the eigenvalues of the Jacobian matrix. If all eigenvalues of the Jacobian matrix have negative real parts, the equilibrium point is asymptotically stable. If at least one eigenvalue has a positive real part, the equilibrium point is unstable. If the Jacobian matrix has eigenvalues with zero real parts (aside from those with negative real parts), the equilibrium point is in a critical state, and its stability cannot be determined solely by the sign of the eigenvalues. By substituting the eight pure strategy points of the game into the linear transformation matrix, the eigenvalue corresponding to the equilibrium points are obtained, as shown in Table 4 below:

**Table 4:** Eigenvalue corresponding to equilibrium points.

Equilibrium point	Jacobian matrix eigenvalues	Stability	Conditions
	*λ*_1_,*λ*_2_,*λ*_3_		
*M*_1_(0,0,0)	$(r_{1}-r_{2})U_{2}-E_{2}w_{2},S_{1}-S_{2}-(P_{1}-P_{2})w_{2},U_{1}-U_{2}-(w_{1}-w_{2})f_{2}$	ESS	①
*M*_2_(1,0,0)	$E_{2}w_{2}-(r_{1}-r_{2})U_{2},U_{1}-U_{2}-(w_{1}-w_{2})f_{1},S_{1}-S_{2}+(E_{1}-E_{2}-P_{1}+P_{2})w_{2}$	ESS	②
*M*_3_(0,1,0)	(*r*_1_−*r*_2_)*U*_2_−*E*_1_*w*_2_, *S*_2_−*S*_1_+(*P*_1_−*P*_2_)*w*_2_, $U_{1}-U_{2}-(w_{1}-w_{2})f_{2}+w_{2}A_{2}-w_{1}A_{1}$	ESS	③
*M*_4_(0,0,1)	$(r_{1}-r_{2})U_{1}-E_{2}w_{1},S_{1}-S_{2}-(P_{1}-P_{2})w_{1}$, *U*_2_−*U*_1_+(*w*_1_−*w*_2_)*f*_2_	ESS	④
*M*_5_(1,1,0)	*E*_1_*w*_2_−(*r*_1_−*r*_2_)*U*_2_, $U_{1}-U_{2}-(w_{1}-w_{2})f_{1}+w_{2}A_{2}-w_{1}A_{1}$, $S_{2}-S_{1}-(E_{1}-E_{2}-P_{1}+P_{2})w_{2}$	ESS	⑤
*M*_6_(1,0,1)	*E*_2_*w*_1_−(*r*_1_−*r*_2_)*U*_1_, *U*_2_−*U*_1_+(*w*_1_−*w*_2_)*f*_1_, $S_{1}-S_{2}+(E_{1}-E_{2}-P_{1}+P_{2})w_{1}$	ESS	⑥
*M*_7_(0,1,1)	(*r*_1_−*r*_2_)*U*_1_−*E*_1_*w*_1_, *S*_2_−*S*_1_+(*P*_1_−*P*_2_)*w*_1_, $U_{2}-U_{1}+(w_{1}-w_{2})f_{2}-w_{2}A_{2}+w_{1}A_{1}$	ESS	⑦
*M*_8_(1,1,1)	*E*_1_*w*_1_−(*r*_1_−*r*_2_)*U*_1_, $U_{2}-U_{1}+(w_{1}-w_{2})f_{1}-w_{2}A_{2}+w_{1}A_{1}$, $S_{2}-S_{1}-(E_{1}-E_{2}-P_{1}+P_{2})w_{1}$	ESS	⑧

Among the equilibrium conditions for the replicator dynamic system involving the e-commerce platform, consumer, and streamer, notable conditions include: ① $(r_{1}-r_{2})U_{2}<E_{2}w_{2},S_{1}-S_{2}<(P_{1}-P_{2})w_{2},U_{1}-U_{2}<(w_{1}-w_{2})f_{2}$; ② $E_{2}w_{2}<(r_{1}-r_{2})U_{2},U_{1}-U_{2}<(w_{1}-w_{2})f_{1},S_{1}-S_{2}<(E_{2}-E_{1}-P_{2}+P_{1})w_{2}$; ③ $E_{2}w_{2}<(r_{1}-r_{2})U_{2},U_{1}-U_{2}<(w_{1}-w_{2})f_{1},S_{2}-S_{1}>(E_{1}-E_{2}-P_{1}+P_{2})w_{2}$; ④ $(r_{1}-r_{2})U_{1}<E_{2}w_{1},S_{1}-S_{2}<(P_{1}-P_{2})w_{1},U_{1}-U_{2}>(w_{1}-w_{2})f_{2}$; ⑤ $E_{1}w_{2}<(r_{1}-r_{2})U_{2},U_{1}-U_{2}<(w_{1}-w_{2})f_{1}-w_{2}A_{2}+w_{1}A_{1},S_{2}-S_{1}<(E_{1}-E_{2}-P_{1}+P_{2})w_{2}$; ⑥ $E_{2}w_{1}<(r_{1}-r_{2})U_{1},U_{1}-U_{2}>(w_{1}-w_{2})f_{1},S_{2}-S_{1}>(E_{1}-E_{2}-P_{1}+P_{2})w_{1}$; ⑦ $(r_{1}-r_{2})U_{1}<E_{1}w_{1},S_{1}-S_{2}>(P_{1}-P_{2})w_{1},U_{1}-U_{2}>(w_{1}-w_{2})f_{2}-w_{2}A_{2}+w_{1}A_{1}$; ⑧ $E_{1}w_{1}<(r_{1}-r_{2})U_{1},U_{1}-U_{2}>(w_{1}-w_{2})f_{1}-w_{2}A_{2}+w_{1}A_{1},S_{2}-S_{1}<(E_{1}-E_{2}-P_{1}+P_{2})w_{1}$. Based on these conditions, it is inferred that the system *M* has four potential evolutionary stable strategies: *M*_2_(1,0,0),*M*_5_(1,1,0),*M*_6_(1,0,1), and *M*_8_(1,1,1). This analysis reveals the complexity and the variety of stable scenarios that can emerge from the strategic interactions in this tripartite evolutionary game.

### 4.4. Stability analysis of equilibrium points in four scenarios

**Fig 3:**
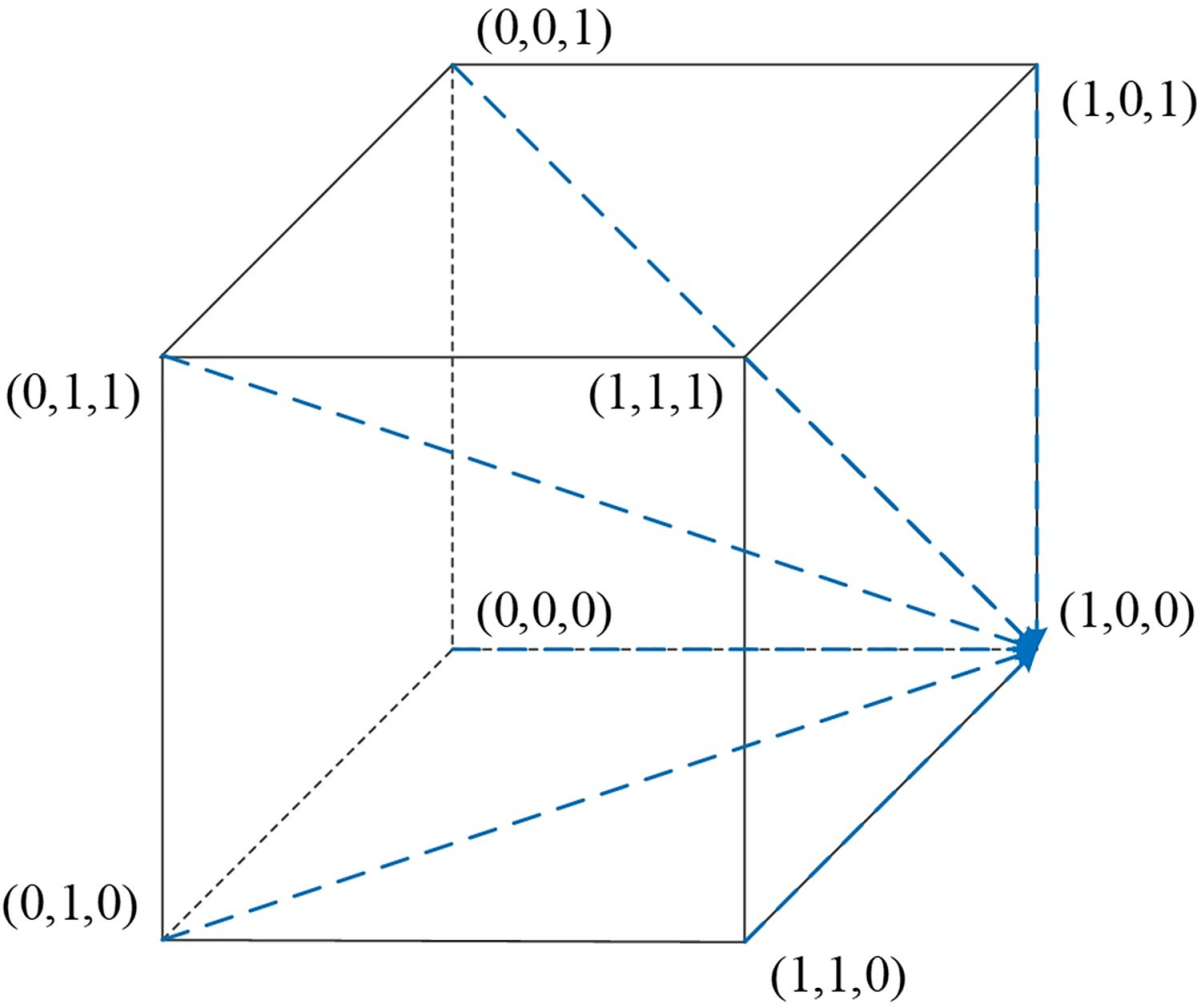
Replicator dynamic phase diagram for scenario 1.

Scenario 1: Under the conditions of ②, specifically when $w_{2}E_{2}<r_{1}U_{2}-r_{2}U_{2},(E_{1}-E_{2}+P_{2}-P_{1})w_{2}<S_{2}-S_{1}$, and *w*_2_*f*_1_−*w*_1_*f*_1_<*U*_2_−*U*_1_, it indicates that when the platform opts for active collaboration and the streamer selects low-quality products, the consumer’s benefits from relaxed requirements are higher than those from strict requirements. Furthermore, when consumers have relaxed requirements, the streamer’s benefits from low-quality selection exceed those from high-quality selection. Also, when consumers have relaxed requirements and the streamer opts for low-quality products, the platform’s benefits from active collaboration are greater than those from passive collaboration. As Table 5 indicates, the Jacobian matrix corresponding to the equilibrium point *M*_2_(1,0,0) has all negative eigenvalues, making *M*_2_(1,0,0) an equilibrium point. Therefore, {Active collaboration, Relaxed requirements, Low-quality selection} constitutes the evolutionary stable strategy in this scenario. The phase diagram of the evolutionary game model for this scenario is shown in Fig 3.

**Table 5:** Stability analysis of equilibrium points in four scenarios.

Equilibrium point		*E*_1_(0,0,0)	*E*_2_(1,0,0)	*E*_3_(0,1,0)	*E*_4_(0,0,1)	*E*_5_(1,1,0)	*E*_6_(1,0,1)	*E*_7_(0,1,1)	*E*_8_(1,1,1)
Scenario 1	*λ*_1_	*+*	−	+	+	−	−	+	−
	*λ*_2_	−	−	+	±	+	±	±	±
	*λ*_3_	−	−	±	+	±	+	±	±
	Stability	Saddle point	ESS	Unstable point	Unstable point	Saddle point	Saddle point	Unstable point	Saddle point
Scenario 2	*λ*_1_	+	−	+	+	−	−	+	−
	*λ*_2_	+	+	−	±	−	±	±	±
	*λ*_3_	−	−	−	+	−	+	+	+
	Stability	Saddle point	Saddle point	Saddle point	Unstable point	ESS	Saddle point	Unstable point	Saddle point
Scenario 3	*λ*_1_	+	−	+	+	−	−	+	−
	*λ*_2_	−	−	+	−	+	−	+	+
	*λ*_3_	±	+	±	±	±	−	±	±
	Stability	Saddle point	Saddle point	Unstable point	Saddle point	Saddle point	ESS	Unstable point	Saddle point
Scenario 4	*λ*_1_	+	−	+	+	−	−	+	−
	*λ*_2_	+	+	−	+	±	+	−	−
	*λ*_3_	+	+	+	−	+	−	−	−
	Stability	Unstable point	Saddle point	Saddle point	Saddle point	Saddle point	Saddle point	Saddle point	ESS

**Note:** In the analysis, "+" indicates a positive eigenvalue for the given scenario, "−" indicates a negative eigenvalue, and "±" indicates that the eigenvalue can be either positive or negative, depending on the scenario.

**Fig 4:**
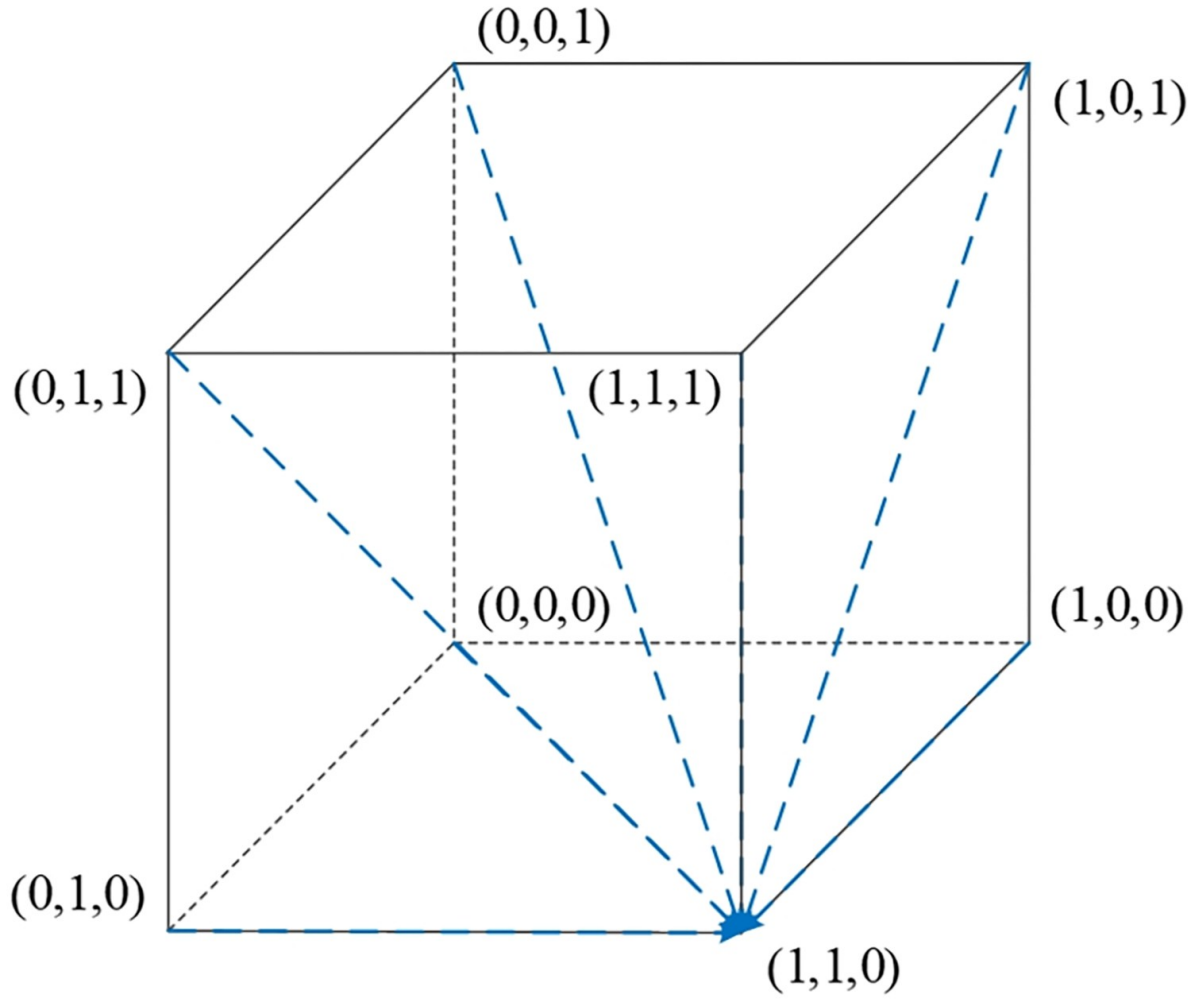
Replicator dynamic phase diagram for scenario 2.

Scenario 2: Meeting conditions of ⑤, namely when $w_{2}E_{1}<r_{1}U_{2}-r_{2}U_{2},(E_{1}–E_{2}+P_{2}–P_{1})w_{2}>S_{2}–S_{1}$, and $w_{2}a_{2}P_{1}-w_{1}a_{1}P_{1}+(w_{2}-w_{1})f_{1}<U_{2}-U_{1}$, indicates that when the platform is actively collaborating and the streamer opts for low-quality selection, the consumers’ gains from strict requirements exceed those from relaxed requirements. Additionally, when consumers have strict requirements, the streamer’s benefits from low-quality selection are greater than from high-quality selection. Also, when consumers have strict requirements and the streamer selects low-quality products, the platform’s benefits from active collaboration surpass those from passive collaboration. As per Table 5, the equilibrium point *M*_5_(1,1,0) has all negative eigenvalues in its Jacobian matrix, thus *M*_5_(1,1,0) is an equilibrium point, and {Active collaboration, Strict requirements, Low-quality selection} is the evolutionary stable strategy in this scenario. The phase diagram for this evolutionary game model is shown in Fig 4.

**Fig 5:**
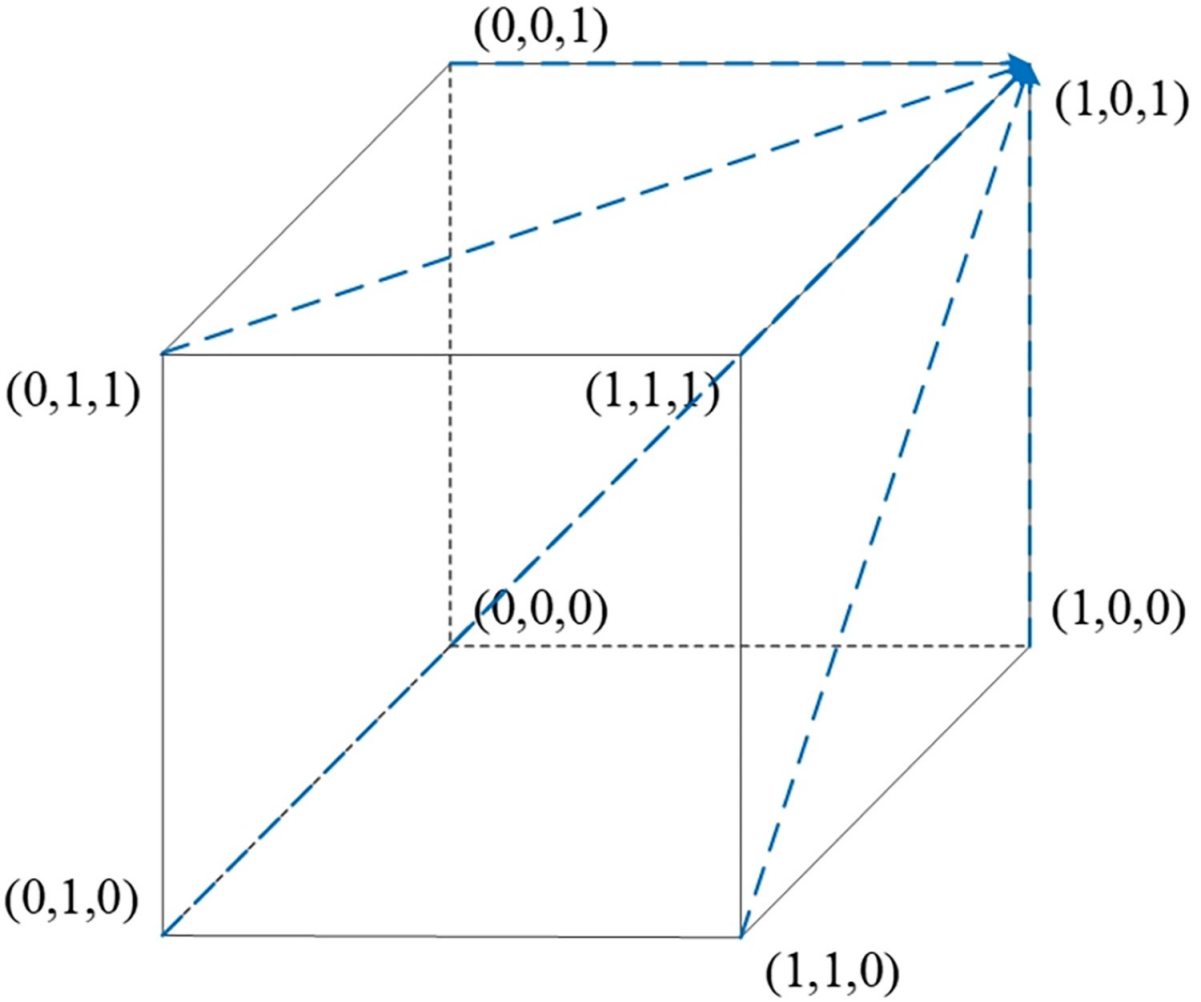
Replicator dynamic phase diagram for scenario 3.

Scenario 3: Fulfilling conditions of ⑥, specifically when $w_{1}E_{2}<r_{1}U_{2}-r_{2}U_{2},(E_{1}-E_{2}+P_{2}-P_{1})w_{1}<S_{2}-S_{1}$, and *w*_2_*f*_1_−*w*_1_*f*_1_>*U*_2_−*U*_1_, suggests that when the platform actively collaborates and the streamer opts for high-quality selection, the benefits for consumers having relaxed requirements exceed those from strict requirements. Moreover, when consumers have relaxed requirements, the streamer’s benefits from high-quality selection are higher than from low-quality selection. Also, when consumers have relaxed requirements and the streamer opts for high-quality products, the platform’s benefits from active collaboration are higher than those from passive collaboration. According to Table 5, the equilibrium point *M*_6_(1,0,1) has all negative eigenvalues in its Jacobian matrix, making *M*_6_(1,0,1) an equilibrium point. Therefore, {Active collaboration, Relaxed requirements, High-quality selection} is the evolutionary stable strategy in this scenario. The phase diagram for this evolutionary game model is illustrated in Fig 5.

**Fig 6:**
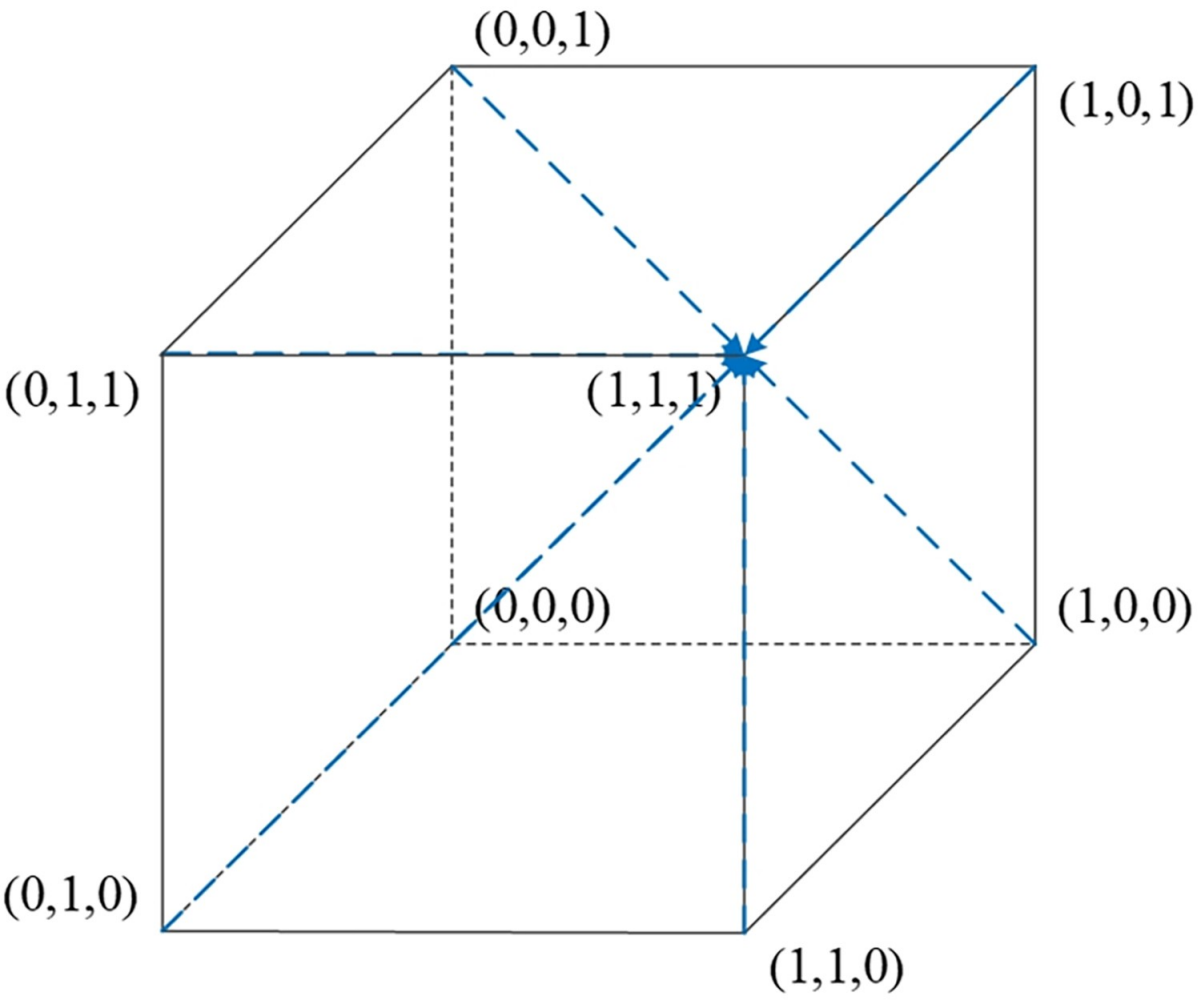
Replicator dynamic phase diagram for scenario 4.

Scenario 4: Fulfilling conditions outlined in ⑧, specifically when $w_{1}E_{1}<r_{1}U_{2}-r_{2}U_{2},(E_{1}-E_{2}+P_{2}-P_{1})w_{1}>S_{2}-S_{1}$, and $w_{2}a_{2}P_{1}-w_{1}a_{1}P_{1}+(w_{2}–w_{1})f_{1}>U_{2}-U_{1}$, suggests that when the platform chooses active collaboration and the streamer opts for high-quality selection, the benefits for consumers adhering to strict requirements exceed those from relaxed requirements. Additionally, when consumers have strict requirements, the streamer’s benefits from high-quality selection are greater than from low-quality selection. Furthermore, when consumers have strict requirements and the streamer opts for high-quality products, the platform’s benefits from active collaboration surpass those from passive collaboration. According to Table 5, the Jacobian matrix eigenvalues corresponding to the equilibrium point *M*_8_(1,1,1) are all negative, making *M*_8_(1,1,1) an equilibrium point. Therefore, (Active Collaboration, Strict Requirements, High-Quality Selection) constitutes the evolutionary stable strategy in this scenario. The phase diagram of the evolutionary game model for this scenario is shown in Fig 6.

The stability of each equilibrium point across these four scenarios is detailed in Table 5. This analysis helps to understand the dynamic interactions and potential outcomes in different strategic combinations within the live streaming e-commerce environment.

## 5. Simulation analysis

The system stability analysis reveals that the relationship between different parameters and variables leads to varying behavioral trends among e-commerce platforms, consumers, and streamers. This section employs Matlab 2022a to conduct evolutionary simulation analysis. Initially, parameters are set based on literature review and empirical investigation. Subsequently, simulation analysis is performed on the evolution of strategies among livestream e-commerce entities. Finally, the impact of parameter changes on the stability of strategy evolution is explored.

### 5.1. Parameter setting

Data plays a crucial role in simulation analysis, where the accuracy and reliability of the analysis need to be verified by comparison with actual data. Utilizing high-quality data helps ascertain the precision of the model and identify any potential biases or errors within it. The strategic evolution of the tripartite game among live streaming platforms, streamers, and consumers in live streaming e-commerce is relatively complex, requiring simulation analysis of four scenarios using Matlab2022a software. General data sets are derived from empirical data [23], real-world examples [23,57], and references [41,58]. The simulation parameters in this paper are set according to the simulation parameter principles found in relevant literature [59,60] and the relevant regulations of the "Consumer Rights Protection Law of the People’s Republic of China". Due to the complexity of the parameters in the model, this paper discusses the stable strategies of evolutionary games under four ideal scenarios. According to the system stability analysis, when the relationship between parameters and variables changes, the decisions of e-commerce platforms, consumers, and streamers also change, exhibiting uncertainty. The values of the simulation parameters are set as shown in Tables 6–9.

**Table 6:** Simulation parameters for scenario 1.

*w*_1_,*w*_2_	*U*_1_,*U*_2_	*r*_1_,*r*_2_	*n*_1_,*n*_2_	*S*_1_,*S*_2_	*P*_1_,*P*_2_	*E*_1_,*E*_2_	*f*_1_,*f*_2_	*a*_1_,*a*_2_
0.2,0.4	8,12	0.6,0.3	8,6	4,3	10,3	3,2	12,10	0.8,0.9

**Table 7:** Simulation parameters for scenario 2.

*w*_1_,*w*_2_	*U*_1_,*U*_2_	*r*_1_,*r*_2_	*n*_1_,*n*_2_	*S*_1_,*S*_2_	*P*_1_,*P*_2_	*E*_1_,*E*_2_	*f*_1_,*f*_2_	*a*_1_,*a*_2_
0.2,0.4	8,12	0.6,0.3	8,6	4,3	7,3	4,2	12,10	0.8,0.9

**Table 8:** Simulation parameters for scenario 3.

*w*_1_,*w*_2_	*U*_1_,*U*_2_	*r*_1_,*r*_2_	*n*_1_,*n*_2_	*S*_1_,*S*_2_	*P*_1_,*P*_2_	*E*_1_,*E*_2_	*f*_1_,*f*_2_	*a*_1_,*a*_2_
0.2,0.4	8,11	0.6,0.3	8,6	4,3	10,3	3,2	16,10	0.8,0.9

**Table 9:** Simulation parameters for scenario 4.

*w*_1_,*w*_2_	*U*_1_,*U*_2_	*r*_1_,*r*_2_	*n*_1_,*n*_2_	*S*_1_,*S*_2_	*P*_1_,*P*_2_	*E*_1_,*E*_2_	*f*_1_,*f*_2_	*a*_1_,*a*_2_
0.2,0.4	8,11	0.6,0.3	8,6	4,3	5,3	3,2	12,10	0.8,0.9

### 5.2. Simulation analysis of strategy evolution in live streaming e-commerce market

**Fig 7:**
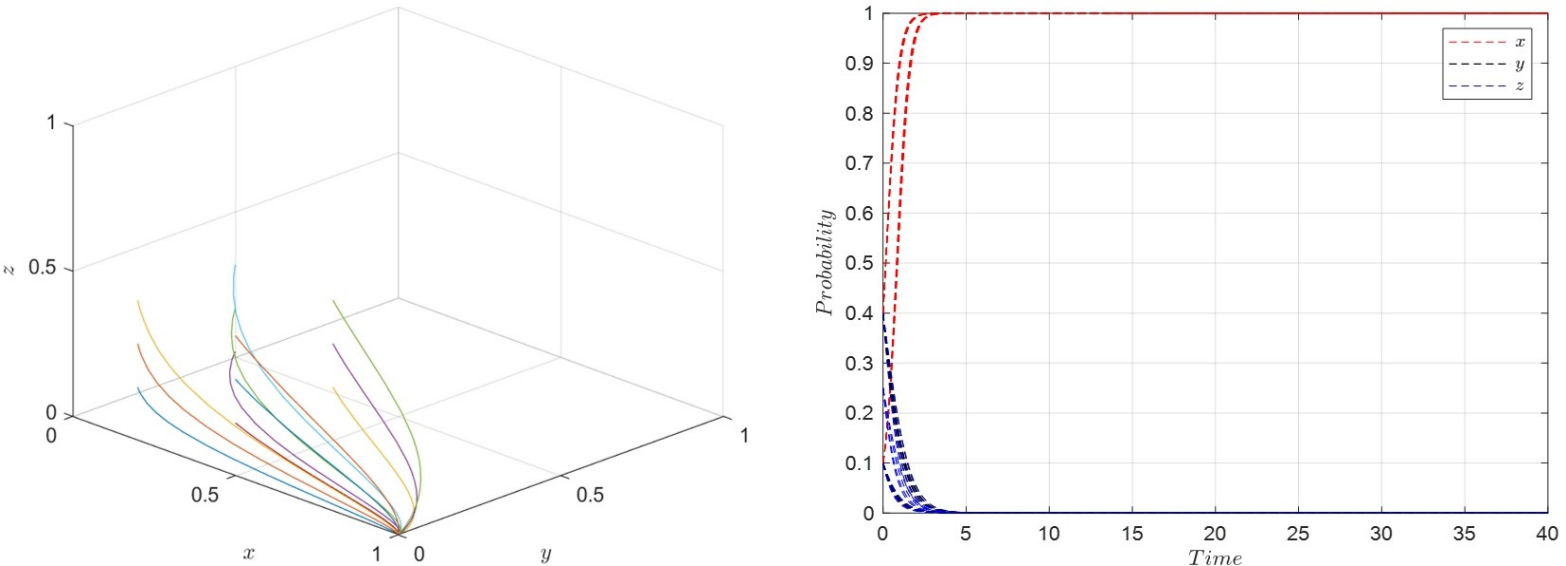
Evolutionary simulation for scenario 1.

Scenario 1: Under the specified conditions ②, namely when $E_{2}w_{2}<(r_{1}-r_{2})U_{2},U_{1}-U_{2}<(w_{1}-w_{2})f_{1}$, and *S*_1_−*S*_2_ < (*E*_2_−*E*_1_−*P*_2_+*P*_1_)*w*_2_, the parameters are set as shown in Table 6, and the simulation results are presented in Fig 7.

Based on the above parameter settings, the process of strategy evolution in the system is depicted in the following Fig 7.

The simulation depicted in Fig 7 indicates that the equilibrium stable strategy *M*_2_(1,0,0), representing {Active collaboration, Relaxed requirements, Low-quality selection}, is prevalent under these conditions. It is observed that when the costs associated with protecting consumer rights are high, it diminishes consumer initiative to defend their rights, leading them to be more lenient in their quality requirements during shopping. In this situation, where consumers are less demanding about quality issues, the e-commerce platform opts for active collaboration. Given the consumers’ low demands and the platform’s weaker punitive measures for quality issues, streamers are more inclined to choose low-quality products. The {Active collaboration, Relaxed requirements, Low-quality selection} becomes the evolutionary stable strategy, and the simulation results of the evolutionary game corroborate the theoretical analysis of Scenario 1.

**Fig 8:**
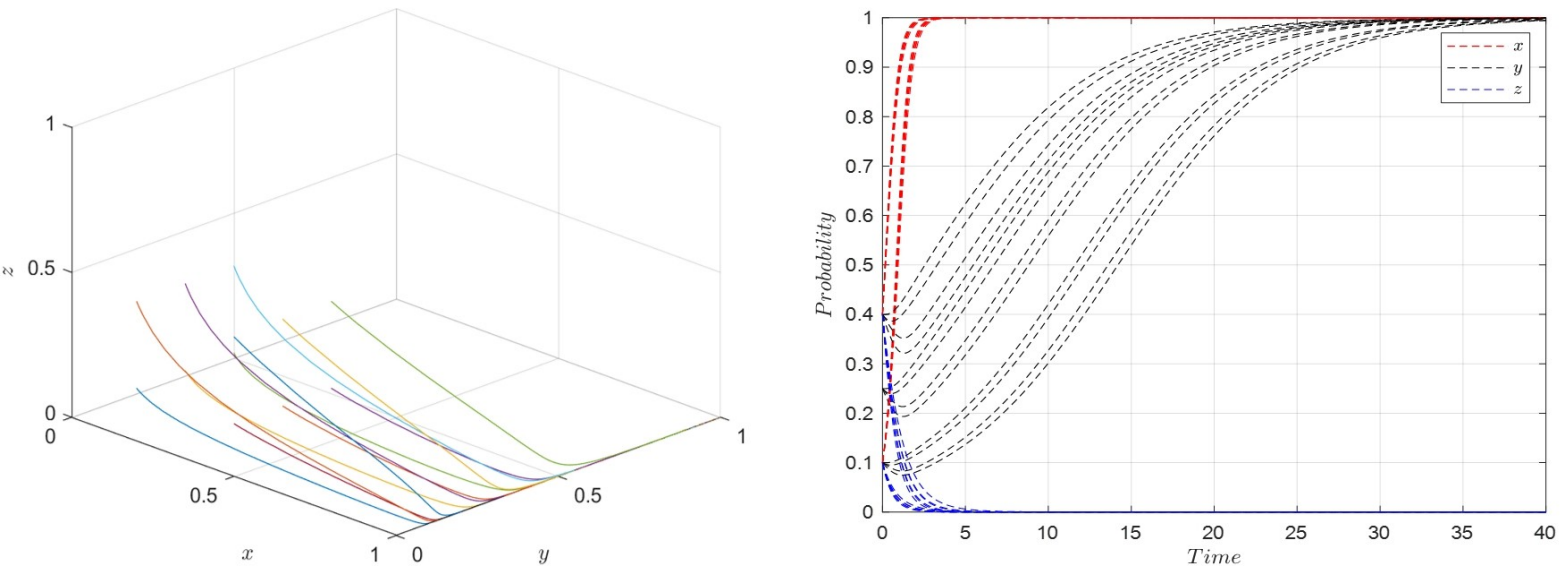
Evolutionary simulation for scenario 2.

Scenario 2: Fulfilling conditions of ⑤, specifically when $E_{1}w_{2}<(r_{1}-r_{2})U_{2},U_{1}-U_{2}<(w_{1}-w_{2})f_{1}-w_{2}A_{2}+w_{1}A_{1}$, and *S*_2_−*S*_1_ < (*E*_1_−*E*_2_−*P*_1_+*P*_2_)*w*_2_, the parameters are set as shown in Table 6, and the simulation results are presented in Fig 8.

The system strategy evolution process based on the above parameters is as shown in Fig 8.

From the simulation shown in Fig 7, it is evident that the equilibrium stable strategy *M*_5_(1,1,0), representing {Active collaboration, Strict requirements, Low-quality selection}, is the prevalent outcome. This suggests that as the costs associated with protecting consumer rights decrease, consumers tend to raise their expectations regarding product quality. Despite the increased costs associated with active collaboration, the combined benefits for the e-commerce platform and streamer from active collaboration still outweigh those from general collaboration, leading the platform to choose active collaboration. However, due to the still weak punitive measures of the platform for quality issues, the cost savings from streamers choosing low-quality products outweigh the losses incurred from consumers’ heightened standards. Therefore, streamers continue to opt for a low-quality selection strategy. The {Active collaboration, Strict requirements, Low-quality Selection} becomes the evolutionary stable strategy, validating the theoretical analysis of Scenario 2 through evolutionary game simulation results.

**Fig 9:**
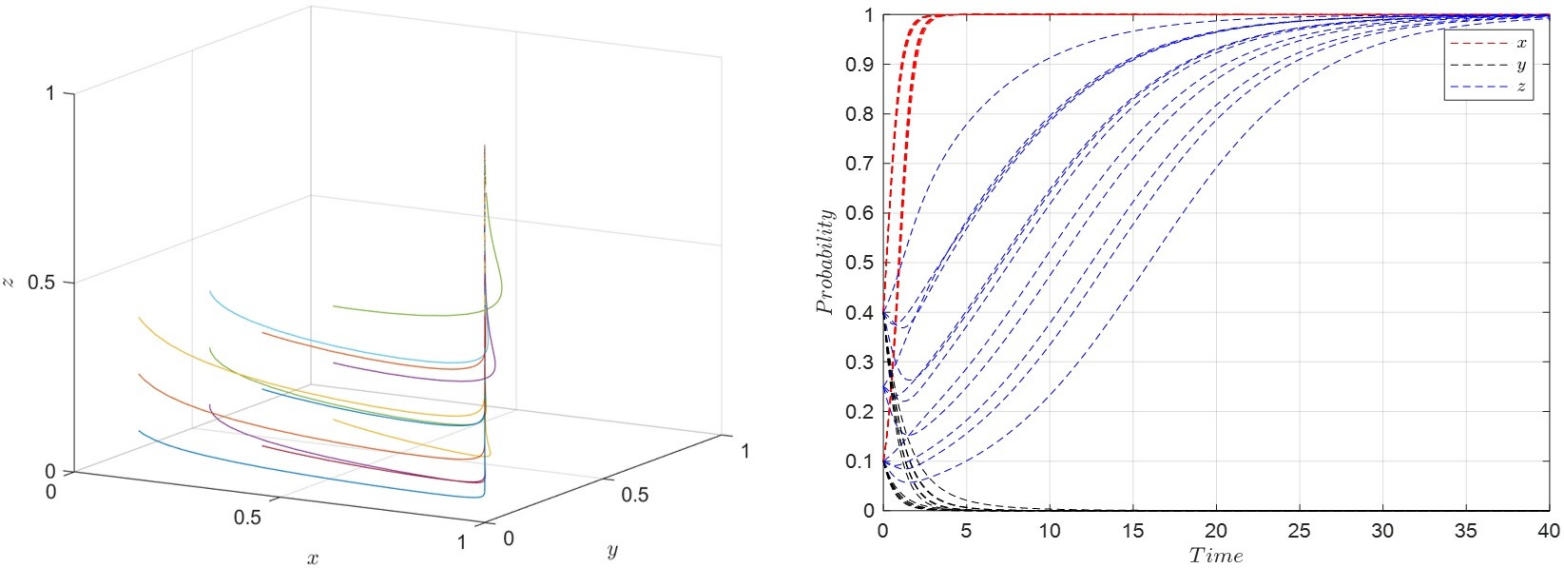
Evolutionary simulation for scenario 3.

Scenario 3: Meeting conditions of ⑥, specifically when $E_{2}w_{1}<(r_{1}-r_{2})U_{2},U_{1}-U_{2}>(w_{1}-w_{2})f_{1}$, and *S*_2_−*S*_1_ = (*E*_1_−*E*_2_−*P*_1_+*P*_2_)*w*_2_, the parameters are set as shown in Table 8, and the simulation results are presented in Fig 9.

Based on the parameter settings mentioned above, the strategy evolution process of the system is illustrated in Fig 9.

The simulation reveals that the equilibrium stable strategy *M*_6_(1,0,1), representing {Active collaboration, Relaxed requirements, High-quality selection}, is the prevalent outcome. This indicates that due to the significant benefits obtained by e-commerce platforms in active collaboration with streamers, platforms often opt for active collaboration. When the costs associated with protecting consumer rights are high, it diminishes consumer initiative to defend their rights, leading them to be more lenient in their quality requirements during shopping. Despite consumers having lower expectations of streamers, the strong punitive measures of the platform for quality issues mean that the gains from streamers choosing low-quality products are insufficient to offset the losses caused by quality problems, naturally inclining streamers to opt for high-quality selections. The {Active collaboration, Relaxed requirements, High-quality selection} becomes the evolutionary stable strategy, validating the theoretical analysis of Scenario 3 through evolutionary game simulation results.

**Fig 10:**
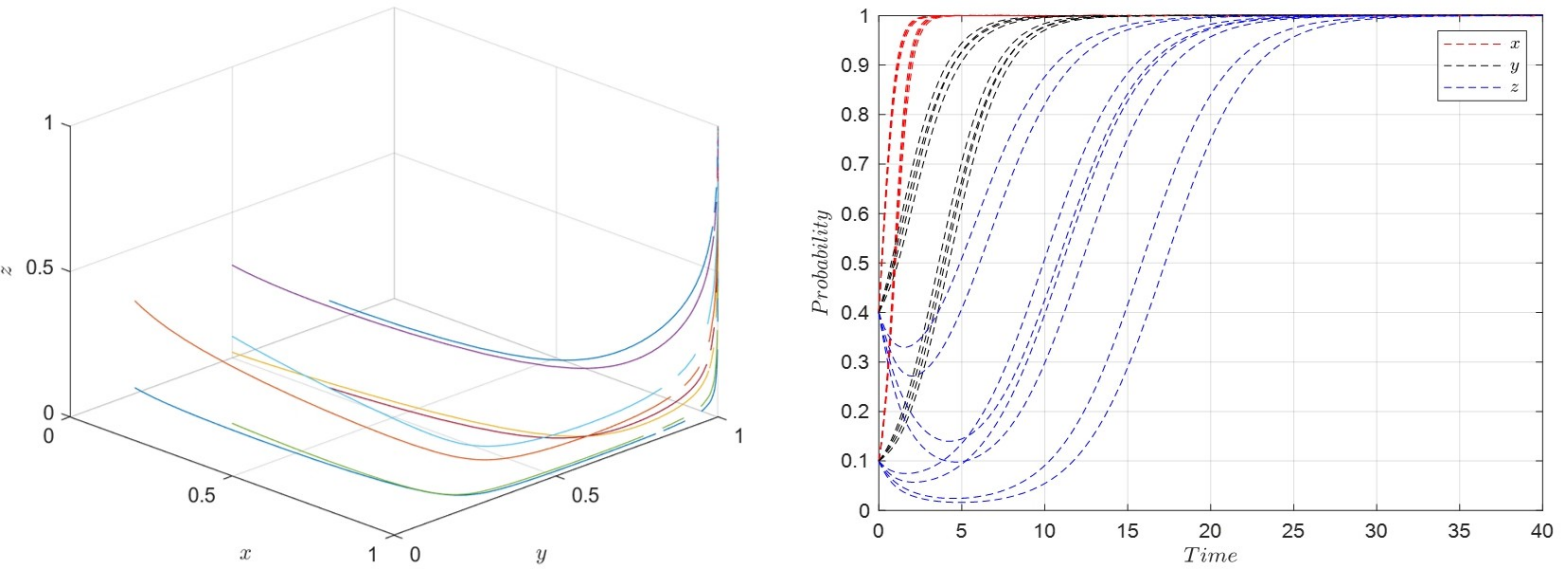
Evolutionary simulation for scenario 4.

Scenario 4: Fulfilling conditions of ⑧, specifically when $E_{1}w_{1}<(r_{1}-r_{2})U_{1},U_{1}-U_{2}>(w_{1}-w_{2})f_{1}-w_{2}A_{2}+w_{1}A_{1}$, and *S*_2_−*S*_1_ < (*E*_1_−*E*_2_−*P*_1_+*P*_2_)*w*_1_, the parameters are set as shown in Table 9, and the simulation results are presented in Fig 10.

Based on the aforementioned parameter settings, the system strategy evolution process is illustrated in Fig 10.

From the simulations, it is evident that the equilibrium stable strategy *M*_8_(1,1,1), representing {Active collaboration, Strict requirements, High-quality selection}, is the prevalent outcome. This scenario reveals that due to significant benefits from active collaboration between e-commerce platforms and streamers, platforms often choose active collaboration. As the costs associated with protecting consumer rights decrease, consumers tend to raise their quality requirements for streamers. In this situation, with consumers imposing strict requirements and platforms exerting strong punitive measures for quality issues, the risk of losses from quality problems is high. Consequently, streamers naturally lean towards reducing the likelihood of quality issues and adopt high-quality selection strategies. The {Active collaboration, Strict requirements, High-quality selection} becomes the evolutionary stable strategy, confirming the validity of the theoretical analysis for Scenario 4 through evolutionary game simulation results.

### 5.3. Impact of parameter on strategy evolution stability

**Fig 11:**
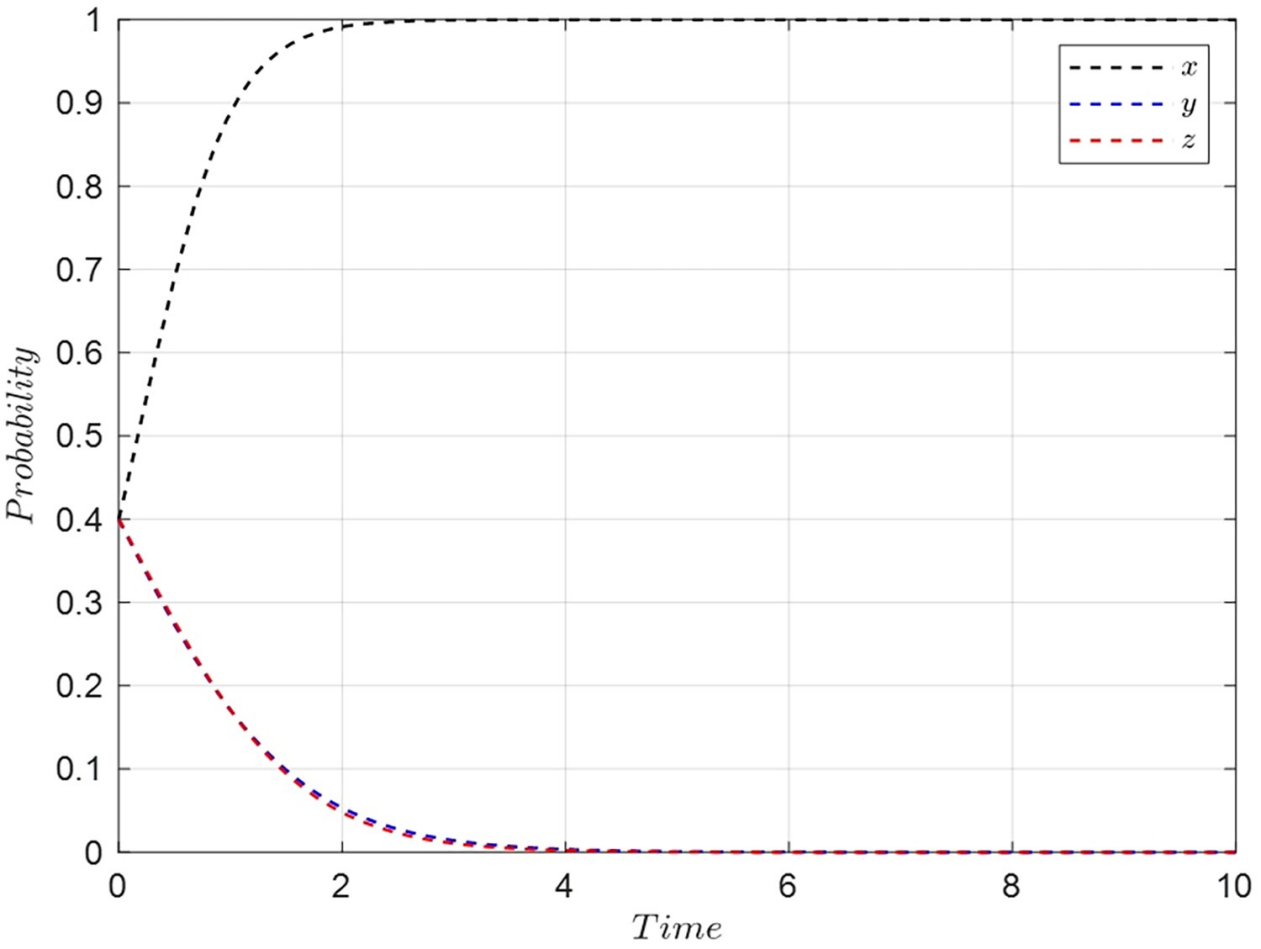
Evolutionary simulation for initial decision *M*_2_(1,0,0).

In the early stages of development for short video and social live streaming e-commerce platforms, e-commerce platforms often chose active collaboration, proactively pushing their original user base towards streamers. At this time, both consumers and streamers were typically in a wait-and-see state. Consequently, consumers had relaxed requirements while watching live streaming, and streamers, seeking to avoid risks and unwilling to invest heavily, opted for low-quality selections. In this scenario, the initial strategies for the e-commerce platforms, consumers, and streamers align with *M*_2_(1,0,0), choosing {Active collaboration, Relaxed requirements, Low-quality selection}. The parameter values are set as previously indicated in Table 6, with the initial probabilities for *x*,*y*, and *z* being set at 0.4. The evolutionary simulation for this decision scenario is presented in Fig 11.

This simulation illustrates how the dynamics of strategy evolution are influenced by initial decisions and parameter settings in the live streaming e-commerce context. It demonstrates the interaction between platform collaboration strategies, consumer requirements, and streamer product selection under specific initial conditions. Understanding these dynamics is crucial for e-commerce platforms, consumers, and streamers to adapt their strategies effectively in response to evolving market conditions and user behavior trends.

#### 5.3.1. Analysis of factors influencing consumer decisions

The transition from *M*_2_(1,0,0) to *M*_5_(1,1,0) represents a shift in consumer decisions from relaxed to strict requirements. Based on the eigenvalues at these equilibrium points, the change in consumer decisions is primarily associated with the parameters *P*_*i*_,*E*_*i*_, and *S*_*i*_. Therefore, the study focuses on the impact of variations in these three parameters. The critical conditions for the transition from *M*_2_ to *M*_5_ are $U_{1}-U_{2}=(w_{1}-w_{2})f_{1}-w_{2}A_{2}+w_{1}A_{1}$ and *S*_2_−*S*_1_ = (*E*_1_−*E*_2_−*P*_1_+*P*_2_)*w*_2_. When *P*_1_≤7, the condition $(w_{2}-w_{1})f_{1}+w_{2}A_{2}-w_{1}A_{1}<U_{2}-U_{1}$ is satisfied, and the critical point becomes only *S*_2_−*S*_1_ = (*E*_1_−*E*_2_−*P*_1_+*P*_2_)*w*_2_.

**Fig 12:**
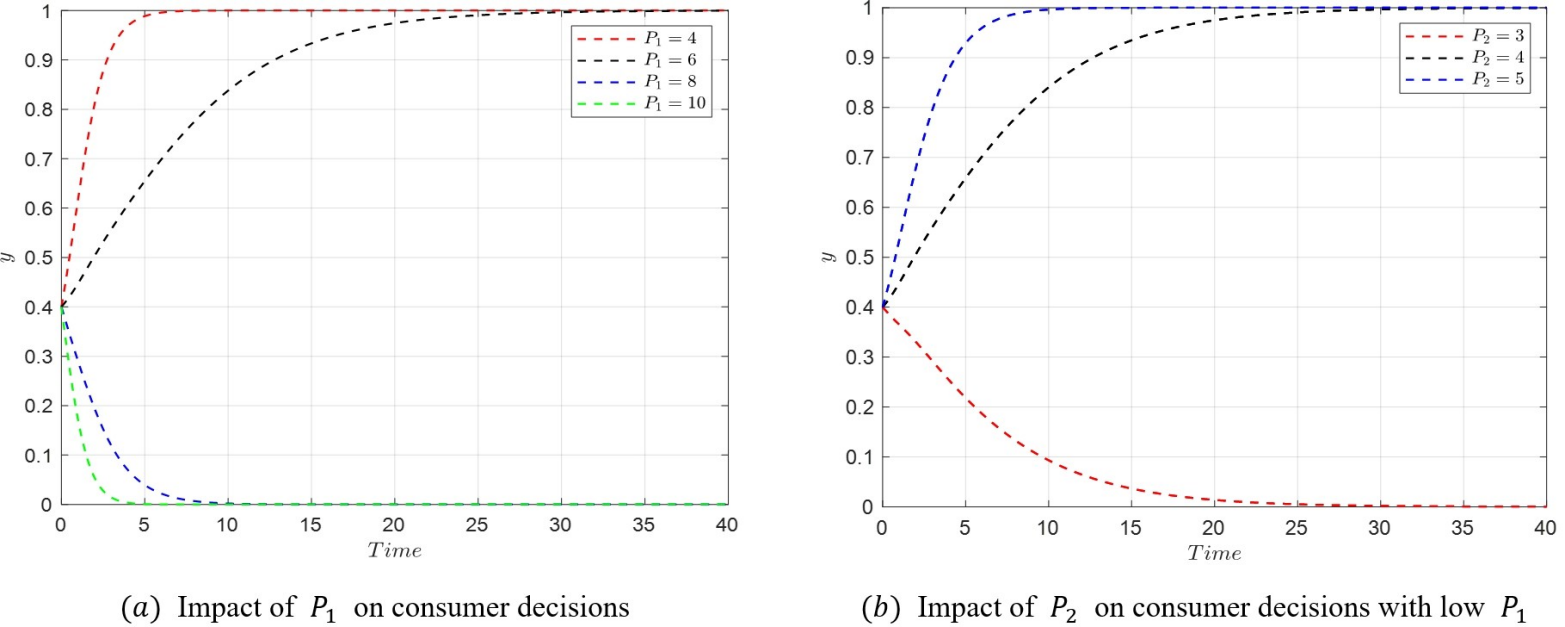
Evolutionary simulation from *M*_2_(1,0,0) to *M*_5_(1,1,0).

**Fig 13:**
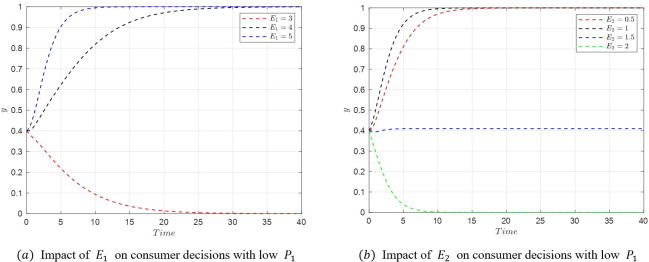
Evolutionary simulation from *M*_2_(1,0,0) tp *M*_5_(1,1,0).

**Fig 14:**
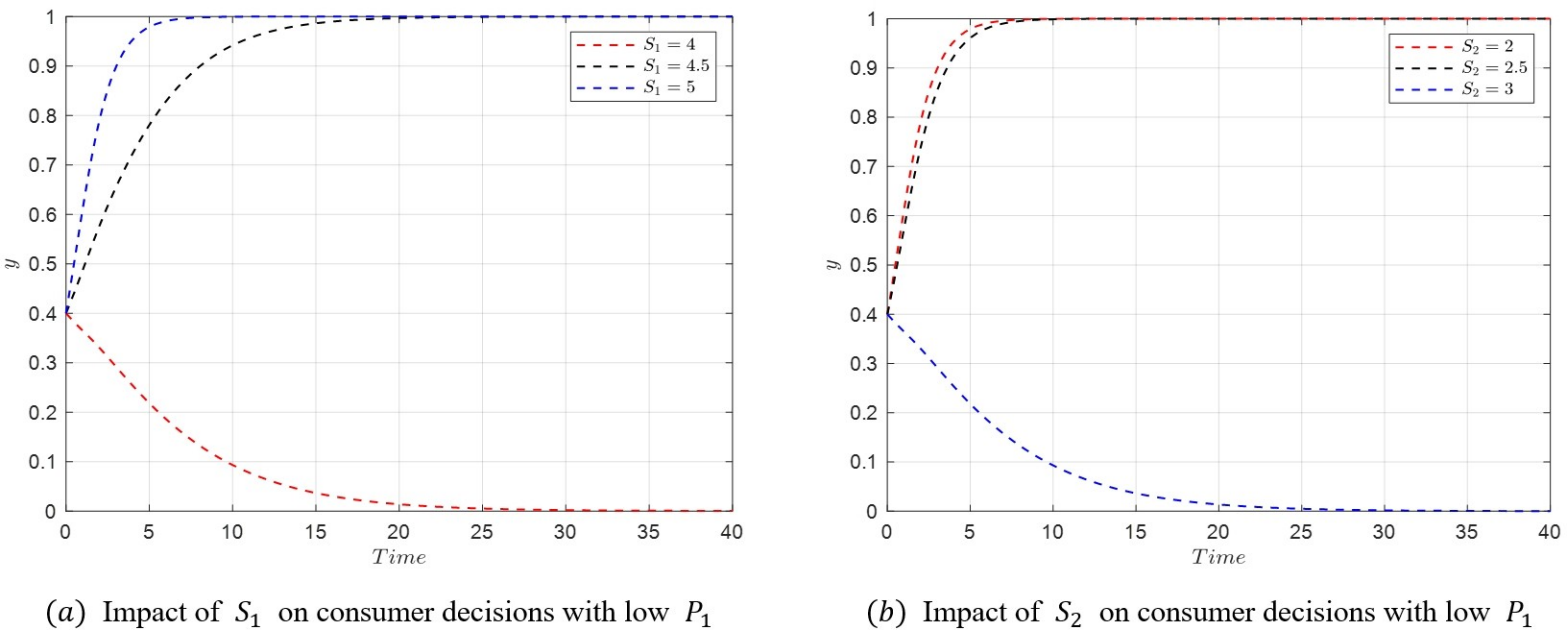
Evolutionary simulation from *M*_2_(1,0,0) to *M*_5_(1,1,0).

(1) When *P*_1_ decreases, satisfying $U_{1}-U_{2}<(w_{1}-w_{2})f_{1}-w_{2}A_{2}+w_{1}A_{1}$ and *S*_2_−*S*_1_ = (*E*_1_−*E*_2_−*P*_1_+*P*_2_)*w*_2_, the stable point transitions from *M*_2_(1,0,0) to *M*_5_(1,1,0) as shown in Fig 12(A). In a low *P*_1_ state (*P*_1_ = 7), when *P*_2_ increases, satisfying *S*_2_−*S*_1_ = (*E*_1_−*E*_2_−*P*_1_+*P*_2_)*w*_2_, the stable point shifts from *M*_2_(1,0,0) to *M*_5_(1,1,0) as depicted in Fig 12(B).

(2) Similarly, in a low *P*_1_ state, when *E*_1_ increases, the stable point transitions from *M*_2_(1,0,0) to *M*_5_(1,1,0), as shown in Fig 13(A). Also, in a low *P*_1_ state, when *E*_2_ decreases, the stable point shifts from *M*_2_(1,0,0) to *M*_5_(1,1,0) as depicted in Fig 13(B). Since *S*_2_−*S*_1_ = (*E*_1_−*E*_2_+*P*_2_−*P*_1_)*w*_2_ is the critical condition for the transition from *M*_2_(1,0,0) to *M*_5_(1,1,0), when *S*_2_−*S*_1_ = (*E*_1_−*E*_2_+*P*_2_−*P*_1_)*w*_2_, both *M*_2_(1,0,0) and *M*_5_(1,1,0) are in a stable state, and consumers can choose either. In such a scenario, the probability of choosing strict requirements y will stabilize but will be neither 1 nor 0. A similar situation was also reported in Liu et al. [61].

(3) In a low *P*_1_ state, when *S*_1_ increases, the stable point shifts from *M*_2_(1,0,0) to *M*_5_(1,1,0), as shown in Fig 14(A). Similarly, when *S*_2_ decreases in a low *P*_1_ state, the stable point transitions from *M*_2_(1,0,0) to *M*_5_(1,1,0) as illustrated in Fig 14(B).

As shown in the figures above, increasing the emotional gains *S*_1_ and compensations *E*_1_ received by consumers for strict requirements can prompt a shift in consumer decision-making from relaxed to strict requirements. Similarly, reducing the cost *P*_1_ of consumer accountability in strict requirement scenarios also leads to a shift from relaxed to strict requirements. Conversely, if the benefits and compensations received under relaxed requirements increase, along with a decrease in the cost of accountability, consumers are likely to revert their decision from strict back to relaxed requirements.

#### 5.3.2. Analysis of factors influencing streamer decisions

The transition from *M*_5_(1,1,0) to *M*_8_(1,1,1) reflects a shift in the streamer’s decision-making from low-quality to high-quality selection. Based on the eigenvalues at these equilibrium points, we can deduce that the changes in the streamer’s decisions are mainly influenced by *U*_*i*_ and *w*_*i*_. Therefore, the study focuses on the impact of these two parameters. Assuming the initial stable point is *M*_5_(1,1,0) with parameter values as shown in Table 7, the critical condition for the transition from *M*_5_(1,1,0) to *M*_8_(1,1,1) is $U_{2}-U_{1}=(w_{2}-w_{1})f_{1}+w_{2}A_{2}-w_{1}A_{1}$.

**Fig 15:**
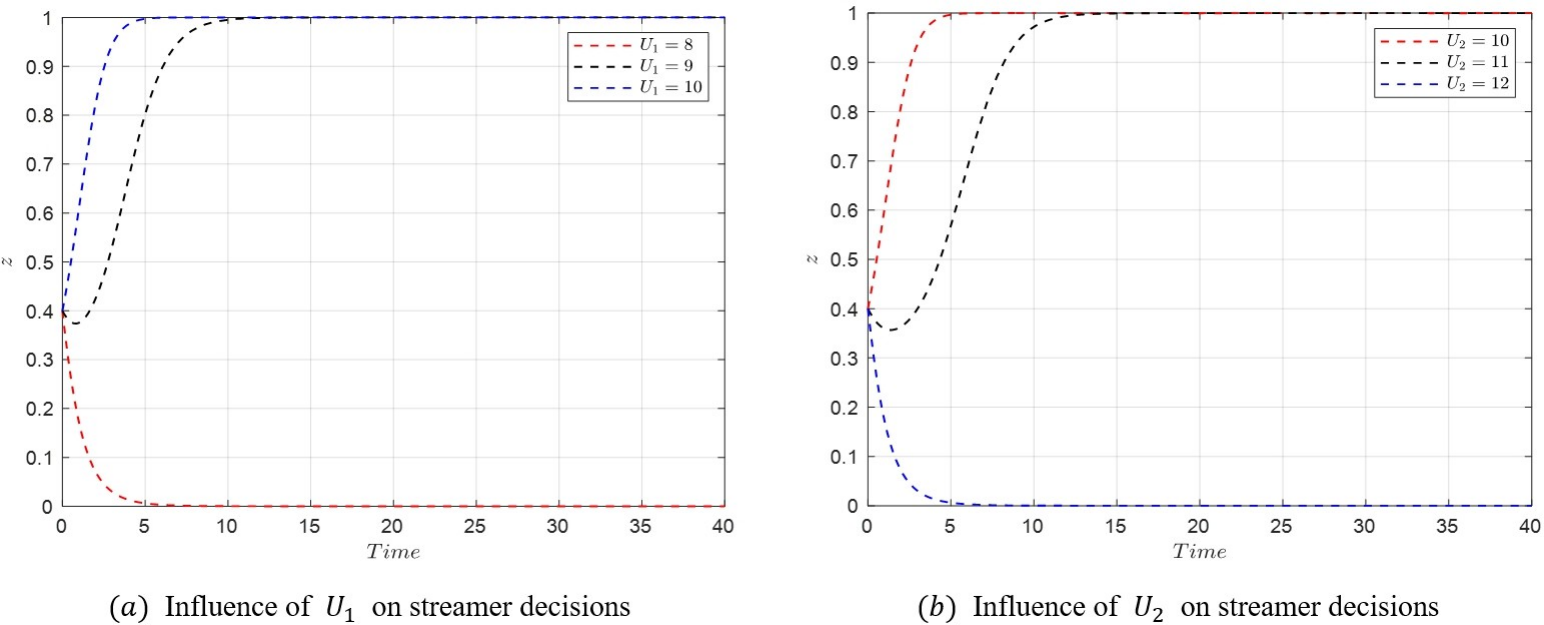
Evolutionary Simulation from *M*_5_(1,1,0) to *M*_8_(1,1,1).

**Fig 16:**
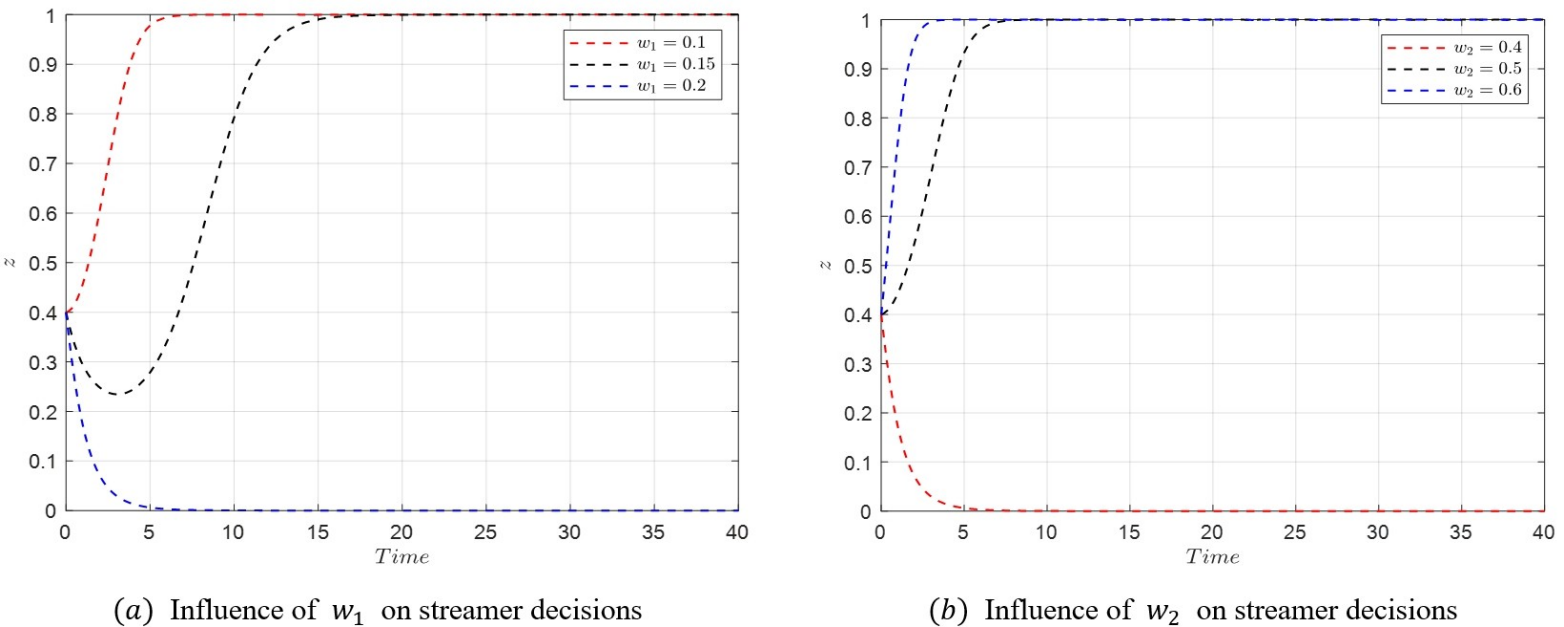
Evolutionary simulation from *M*_5_(1,1,0) to *M*_8_(1,1,1).

(1) When *U*_1_ increases or *U*_2_ decreases, satisfying $U_{2}-U_{1}<(w_{2}-w_{1})f_{1}+w_{2}A_{2}-w_{1}A_{1}$, the stable point shifts from *M*_5_(1,1,0) to *M*_8_(1,1,1) as shown in Fig 15(A) and 15(B).

(2) Similarly, when *w*_1_ decreases, the stable point shifts from *M*_5_(1,1,0) to *M*_8_(1,1,1) as shown in Fig 16(A). Conversely, when *w*_2_ increases, the stable point transitions from *M*_5_(1,1,0) to *M*_8_(1,1,1) as depicted in Fig 16(B).

The simulations above reveal that the decision-making of streamers is positively correlated with the rewards *U*_*i*_ associated with different selection strategies and negatively correlated with the probability *w*_*i*_ of product issues under each selection strategy. Given that the probability of quality issues is higher with low-quality selection, streamers are inclined to choose high-quality products to minimize penalties. Conversely, if the platform’s regulatory measures are weak, streamers may opt for low-quality selections to maximize profits. This underscores the critical role of platform regulations and the impact of potential rewards and penalties in shaping streamer behavior in the live streaming e-commerce environment.

#### 5.3.3. Analysis of factors influencing e-commerce platform decisions

**Fig 17:**
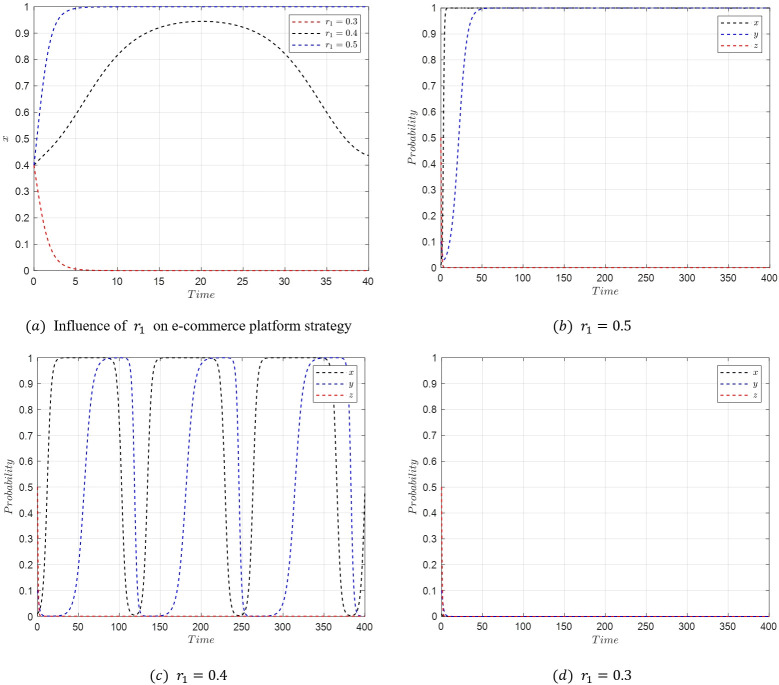
Evolutionary simulation from *M*_5_(1,1,0) to *M*_1_(0,0,0).

In the initial stages of live streaming e-commerce development, with streamers choosing low-quality selections, the entire process of the evolutionary game is studied. Assuming the initial stable point is *M*_5_(1,1,0) with parameters as specified in Table 7, a temporary and unstable emergence of *M*_3_(0,1,0) occurs when the active collaboration reward coefficient *r*_1_ of the platform is reduced to 0.4. As *r*_1_ is further decreased to 0.3, the evolutionary stable point bypasses *M*_3_(0,1,0) and directly transitions to *M*_1_(0,0,0). The strategy evolution process of the system under these conditions is depicted in Fig 17.

Fig 17 demonstrates that when the active collaboration reward coefficient *r*_1_ for the e-commerce platform is high, the platform tends to choose active collaboration. However, as *r*_1_ decreases, a sequence of transitions in the stability of the system is observed. Initially, a reduction in *r*_1_ leads to a change in the stable point from *M*_5_(1,1,0) to *M*_3_(0,1,0), as the profits for the platform under *M*_5_(1,1,0) and *M*_3_(0,1,0) conditions $r_{1}U_{2}-w_{2}n_{1}-w_{2}E_{1}<r_{2}U_{2}-w_{2}n_{1}$ become less favorable. Subsequently, the stable point shifts from *M*_3_(0,1,0) to *M*_1_(0,0,0) as the consumer benefits under *M*_3_(0,1,0) and *M*_1_(0,0,0) conditions *S*_1_−*w*_2_*P*_1_<*S*_2_−*w*_2_*P*_2_ also decrease. Then, as $r_{2}U_{2}-w_{2}n_{2}<r_{1}U_{2}-w_{2}n_{2}-w_{2}E_{2}$, the stable point moves from *M*_1_(0,0,0) to *M*_2_(1,0,0). Finally, the condition $S_{2}-w_{2}P_{2}+w_{2}E_{2}<S_{1}-w_{2}P_{1}+w_{2}E_{1}$ causes the stable point to shift back from *M*_2_(1,0,0) to *M*_5_(1,1,0), creating a cyclical pattern.

When *r*_1_ is further reduced to 0.3 or lower, the final evolutionary stable strategy becomes *M*_1_(0,0,0), characterized by passive collaboration from the e-commerce platform, relaxed consumer requirements, and low-quality selection by streamers. This state fails to stimulate active participation among the system’s stakeholders, potentially leading the live streaming e-commerce market into a low-end and chaotic state. This underscores the crucial role of maintaining appropriate incentives and collaboration levels to foster a healthy and dynamic market environment.

**Fig 18:**
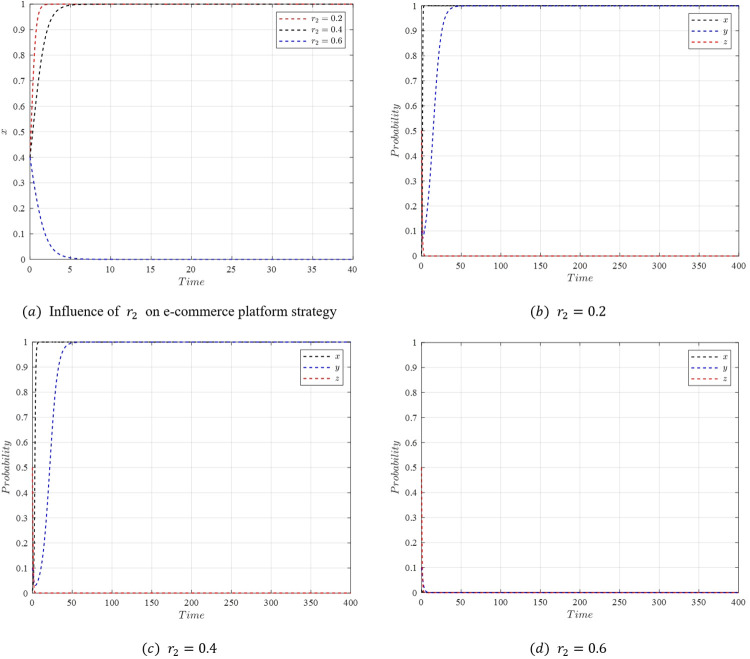
Evolutionary simulation from *M*_5_(1,1,0) to *M*_1_(0,0,0).

Similarly, as shown in Fig 18, when *r*_2_ increases to 0.6 or higher, the final evolutionary strategy also becomes *M*_1_(0,0,0), characterized by general cooperation of e-commerce platforms, general consumer requirements, and general product selection by streamers. This state fails to stimulate active participation from system stakeholders, potentially leading the live streaming e-commerce market into a low-end and disordered state, which is not conducive to maintaining a healthy shopping environment. This highlights the importance of the profit coefficient of e-commerce platforms to the entire evolutionary system.

Simultaneously, the reductions in *r*_1_ and *r*_2_ correspond to the intense competition among e-commerce platforms observed in real life. When *r*_1_ decreases, the strategic choice of e-commerce platforms shifts from active cooperation to general cooperation, whereas a reduction in *r*_2_ sways the platforms’ decision-making towards active cooperation. Moreover, the decrease in *r*_1_ and *r*_2_ also leads to streamers opting for general product selections, and the ultimate market outcome is that both reductions further diminish the profits of e-commerce platforms.

## 6. Conclusion

This study focuses on the increasingly popular trend of live streaming selling, with particular attention to product quality and after-sales issues within this domain. It constructs an evolutionary game model involving e-commerce platforms, consumers, and streamers in live streaming selling. The research analyzes the strategy choices of these parties, the stability of equilibrium strategy combinations in the game system, and the impact of various elements on decision-making behavior. The validity of the analytical conclusions is further substantiated through simulation analysis.

This study delves into the evolutionary game model involving three key participants in live streaming selling and explores evolutionarily stable strategies across four scenarios. It provides explanations for these strategies within the context of live streaming e-commerce and uses simulation analysis to model the stability of equilibrium points under various conditions, also examining the impact of parameter changes on the stability of strategy evolution. The research yields several important conclusions: Firstly, for consumers, measures such as lowering the costs associated with enforcing strict quality requirements or increasing the repercussions when relaxed standards lead to quality issues, as well as enhancing compensations and benefits received under both strict and relaxed requirements, are found to be effective in encouraging consumers to adopt stricter standards. Secondly, for streamers, the study identifies that increasing the rewards for selecting high-quality products and reducing the probability of encountering quality issues with such selections, or conversely, lowering the benefits and increasing the risks associated with low-quality selections, can effectively guide and incentivize streamers towards higher quality offerings. Lastly, the analysis for e-commerce platforms reveals that excessively reducing the profit-sharing in active collaborations can significantly weaken their motivation for such partnerships. This reduction in motivation adversely impacts both consumers and streamers, potentially leading the live streaming selling market into a dilemma akin to a prisoner’s dilemma and resulting in a tragic market scenario.

In response to the study’s insights, several management strategies are recommended for improving the live streaming selling environment. Consumers should maintain rationality while engaging with streamers to avoid impulsive buying influenced by undue trust. They need to be proactive in protecting their rights, seeking after-sales service for any product quality issues and escalating to complaints or reports when necessary. E-commerce platforms hosting these sessions should rigorously monitor product quality and establish robust systems to regulate streamer behavior, including public actions against streamers who violate quality standards and implementing strict measures like reduced exposure or account suspension for serious or repeated infractions. For streamers, particularly celebrity streamers, prioritizing product quality is crucial to safeguard their reputation and credibility. They should ensure all promotions are genuine and lawful, and be prepared to assist with after-sales issues. Brands venturing into live streaming selling must carefully select streamers whose audience aligns with their product positioning and brand ethos to avoid ineffective collaborations.

This study provides valuable insights into live streaming e-commerce, while also highlighting areas ripe for future exploration. Firstly, the current research centers primarily on e-commerce platforms, consumers, and streamers, laying a foundational understanding of this domain. However, future analyses that include additional stakeholders, such as suppliers, could enrich the study due to their crucial role in the live streaming e-commerce ecosystem. Secondly, an exploration of the competitive dynamics between e-commerce platforms and among streamers, coupled with an in-depth examination of the strategies of businesses involved in live streaming e-commerce and the complex interactions in environments with multiple platforms or streamers, could offer a more detailed market understanding. Lastly, investigating areas such as the impact of technological advancements (e.g., artificial intelligence, virtual reality) on live streaming e-commerce or the influence of social media influencers in shaping consumer perceptions constitutes important directions for future research. These areas promise to expand upon the existing research base and furnish a comprehensive view of the evolving theoretical framework of the live streaming e-commerce sector.
